# Evaluating High-Variance Leaves as Uncertainty Measure for Random Forest Regression

**DOI:** 10.3390/molecules26216514

**Published:** 2021-10-28

**Authors:** Thomas-Martin Dutschmann, Knut Baumann

**Affiliations:** Institute for Medicinal and Pharmaceutical Chemistry, University of Technology Braunschweig, Beethovenstraße 55, 38106 Braunschweig, Germany; t.dutschmann@tu-braunschweig.de

**Keywords:** chemoinformatics, machine learning, Random Forest, regression, ensemble, uncertainty measure, reliability measure

## Abstract

Uncertainty measures estimate the reliability of a predictive model. Especially in the field of molecular property prediction as part of drug design, model reliability is crucial. Besides other techniques, Random Forests have a long tradition in machine learning related to chemoinformatics and are widely used. Random Forests consist of an ensemble of individual regression models, namely, decision trees and, therefore, provide an uncertainty measure already by construction. Regarding the disagreement of single-model predictions, a narrower distribution of predictions is interpreted as a higher reliability. The standard deviation of the decision tree ensemble predictions is the default uncertainty measure for Random Forests. Due to the increasing application of machine learning in drug design, there is a constant search for novel uncertainty measures that, ideally, outperform classical uncertainty criteria. When analyzing Random Forests, it appears obvious to consider the variance of the dependent variables within each terminal decision tree leaf to obtain predictive uncertainties. Hereby, predictions that arise from more leaves of high variance are considered less reliable. Expectedly, the number of such high-variance leaves yields a reasonable uncertainty measure. Depending on the dataset, it can also outperform ensemble uncertainties. However, small-scale comparisons, i.e., considering only a few datasets, are insufficient, since they are more prone to chance correlations. Therefore, large-scale estimations are required to make general claims about the performance of uncertainty measures. On several chemoinformatic regression datasets, high-variance leaves are compared to the standard deviation of ensemble predictions. It turns out that high-variance leaf uncertainty is meaningful, not superior to the default ensemble standard deviation. A brief possible explanation is offered.

## 1. Introduction

Computational methods to predict molecular properties play a crucial role in the early stages of drug development, since their estimations determine the following experiments. An insufficient validation of predictive models may lead to wrong assumptions and, therefore, bears the risk of wasting time and resources [1]. Typically, the predictive performance of machine learning models is evaluated using an external test set [2]. However, such evaluations do not reflect the limitations of the model [3]. These limitations partially arise from the training data the model has learned from. Test data that are similar to the training data are expected to be easily predictable [4]. This expectation can be deceptive, since parts of the chemical space that are well covered by the training data can still be hard to model, e.g., in the case of unusually high output fluctuations in dense areas. In quantitative structure–activity relationship (QSAR) research, such areas are known as activity cliffs. Formally, they are pairs or groups of compounds that are structurally similar but differ vastly in their potency [5]. Although they are interesting from a pharmacodynamical point of view, they add to the difficulty of predictive modeling. It is, therefore, desirable to provide an uncertainty parameter additional to each prediction that estimates the reliability of the predicted output [6], e.g., by error bars [7]. Quantifying the uncertainty of predictive models in drug design and chemoinformatics is considered part of assessing their applicability domain. Due to the established, yet still increasing, usage of machine learning in drug design [8,9], exploring and evaluating the applicability domain and uncertainty quantification methods is an on-going field of research [10,11,12,13,14,15,16]. Some modeling techniques must be augmented or modified to yield uncertainty measures (UMs), others have built-in measures due to their operating principle.

Random Forests (RFs) are widely applied machine learning techniques that already provide a UM through construction [17]. An RF consists of individual decision tree estimators that are independently fitted models. According to a given loss function that penalizes errors, a tree learns by finding optimal splitting points for individual independent variables to partition the training data such that the resulting error term becomes minimal. To build an RF, several decision trees are fitted to different randomly chosen subsets of the training data. When growing a tree, the split function chooses the best independent variable and the best splitting point for that variable, but at each step it receives only a randomly selected subset of the variables. These perturbations render the trees in the RF diverse and improve generalization. By default, RFs for regression output the arithmetic mean over all single-tree predictions. Due to the approach of aggregating multiple weak learners to a stronger single predictor, RFs are, by definition, ensemble predictors [18]. Uncertainty estimates for new objects can, in general, be obtained from ensembles by considering the disagreement of the ensemble members’ predictions, where the degree of disagreement is assumed to be related to the difficulty to predict the output. The standard deviation or variance are straight-forward measures to capture how wide the single predictions are distributed around the mean. Measures of prediction distribution are the default uncertainty estimators of ensemble-based methods [19].

Depending on the task at hand, decision trees might be outperformed by other machine learning techniques, but their partitioning strategy is easy to interpret and gives rise to a novel UM. As the trees in an RF focus on local error minimization, their leaves represent areas where the output variance is at a supposed minimum. Thus, more variance in a leaf implies that the local area must be hard to model and objects that are located in that area are, therefore, predicted with more uncertainty. An illustrative example of a high-variance leaf is provided in Figure S1 in the Supporting Information. The concept of uncertainty estimation by screening for high local output variances has already been successfully applied in a model-independent approach [20]. High-variance leaves (HVLs) can be determined by providing a threshold value, where leaves with an output standard deviation above this threshold are HVLs. RF predictions, for which more tree estimators predict from HVLs, can then be considered less certain. The fraction of trees that predicts from HVLs serves as a UM for RF regression. Originally, the idea of HVLs with a threshold was inspired by the discrete definition of activity cliffs, since they imply structurally similar molecules with dissimilar activities [21]. While the standard deviation of ensemble predictions (SDEP) can become infinitely large in theory, the fraction of HVLs lies between 0 and 1 by construction and is, therefore, comparable across datasets. The disadvantage is the requirement of the threshold parameter for the standard deviations in the leaves. The best threshold for the dataset at hand can be found by grid-search or provided by the user from experience. For brevity, the fraction of HVL predictions and the actual HVLs are abbreviated as HVLs.

On a selection of chemoinformatic regression datasets, predominantly target activities, the HVLs are evaluated for their ability to reflect prediction uncertainties. Basic RF models are computed for all datasets and two different descriptors, followed by assessing their predictive quality. HVLs are defined using a constant threshold to give an impression of their base performance, without any additional tuning. For the predictions of each run, the SDEP and HVLs are compared by measuring the decline in error when removing those predictions of highest uncertainty. As expected, HVLs are indeed related to modeling difficulty and perform comparably yet slightly inferior to the SDEP.

## 2. Results

### 2.1. Predictive Performance

The model performances for each dataset are listed in Table 1. Overall, models using ECFPs achieved higher predictive performances. In only 9 out of the 32 datasets, the predictive performance was higher when using RDKit descriptors. For dataset P04150, the models of both descriptor types achieved the same prediction quality.

The three non-bioactivity datasets and Q16602 exhibited the lowest errors among all RDKit descriptor evaluations. The four best-performing datasets for ECFPs were P42345, Q16602, P24530, and P25929. The lowest performances among RDKit descriptor evaluations were obtained for P41594, MMP2, P61169, and O60674. For ECFP evaluations, the datasets Q05397, P28482, P41594, and P28335 had the lowest prediction quality.

The degree of overfitting varied for each combination. While all train $R^{2}$s were located between 0.88 and 0.98, test $R^{2}$s ranged from 0.47 to 0.91. Test performances of the activity datasets were consistent with the results of Cortes-Ciriano [22]. Observed vs. predicted scatter plots for all evaluations give a visual overview of the predictive performances and are provided in Tables S1 and S2 in the Supporting Information.

### 2.2. Area under the Confidence–Oracle Error Curve after Removing 50% of the Most Uncertain Predictions ($AUCO_{50}$)

**Figure 1:**
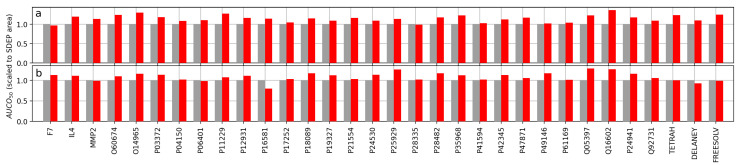
Comparison of the two UMs by $AUCO_{50}$. All values were divided by the $AUCO_{50}$ of the SDEP to show relative areas. The height of each bar indicates the area between ideal curve and that of the measure (smaller is better). Bars corresponding to the SDEP are shown in gray, while bars of HVLs are colored red. The results are shown for all datasets (**a**) when using RDKit descriptors and (**b**) when using ECFPs.

**Figure 2:**
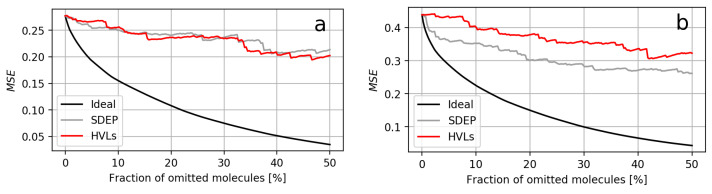
Two confidence curve plots from models using RDKit descriptors. (**a**) Plot of dataset F7, for which HVLs had the smallest area compared to that of the SDEP. (**b**) Plot of dataset Q16602, for which HVLs had the largest area compared to that of the SDEP.

**Figure 3:**
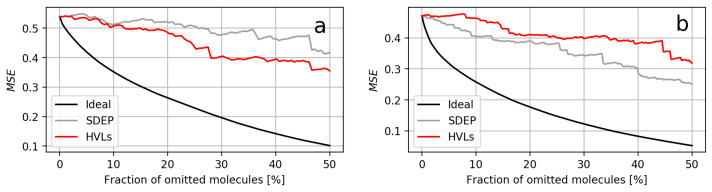
Two confidence curve plots from models using ECFPs. (**a**) Plot of dataset P16581, for which HVLs had the smallest area compared to that of the SDEP. (**b**) Plot of dataset Q05397, for which HVLs had the largest area compared to that of the SDEP.

All comparisons by relative $AUCO_{50}$ (i.e., the $AUCO_{50}$ using HVLs relative to using the SDEP) are shown in Figure 1. When using RDKit descriptors, the areas for the HVLs ranged from 97.4% to 136.9% of the corresponding SDEP area, 114.9% on average. The two extreme cases corresponded to the datasets F7 and Q16602, respectively. For ECFPs, the areas ranged from 80.1% to 129.0%, with 108.7% on average. The extreme cases correspond to the datasets P16851 and Q05397, respectively. The confidence curves for RDKit descriptors are shown in Figure 2, while those for ECFPs are depicted in Figure 3. These confidence curve plots also illustrate how the areas were obtained: The $AUCO_{50}$ for the SDEP arose from the area between the black ideal curve and the gray SDEP confidence curve, and the $AUCO_{50}$ for HVLs arose from the area between the black ideal curve and the red HVLs confidence curve.

**Figure 4:**
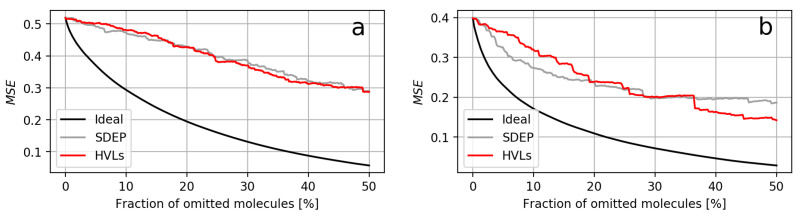
Two confidence curve plots from models using RDKit descriptors. (**a**) Plot of dataset P28335. (**b**) Plot of dataset P49146. Although the area ratios of both UMs in (**a**,**b**) were close to 1, the confidence curves of the HVLs differed in their shape. In (**a**), both curves progressed in a similar fashion, while in (**b**), the SDEP curve declined faster until the curve of the HVLs sharply decreased after 35% and continued to decline.

The datasets where both measures exhibited the most similar areas were P28335 when using RDKit descriptors and TETRAH in case of ECFPs. Similar areas occurred when either the confidence curves of both measures constantly overlaped or one curve crossed the other, such that their areas became alike in the long run. For both possibilities, respectively, the confidence curves of P28335, using RDKit descriptors, and P49146, using RDKit descriptors, are visualized in Figure 4. All confidence curves can be found in Tables S3 and S4 in the Supporting Information. Furthermore, UM vs. residual scatter plots are provided in Tables S5 and S6.

For the comparisons between the SDEP and HVLs involving RDKit descriptors, 22 out of all 32 cases differed more than 10% in their $AUCO_{50}$. Nine cases differed more than 20% and two cases differed more than 30%. For the evaluations when using ECFPs, 18 out of the 32 cases differed more than 10% in $AUCO_{50}$. Four cases differed more than 20%, and no case differed 30% or more.

**Figure 5:**
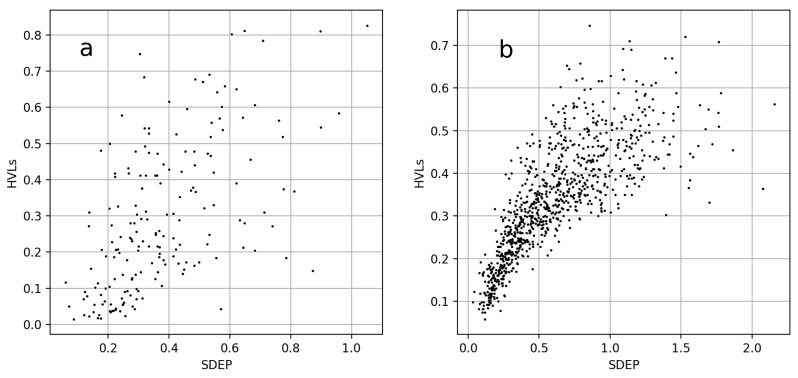
Scatter plots of SDEP vs. HVLs. The SDEP of each object in a dataset was plotted against its fraction of HVLs. (**a**) Plot of dataset P16581, using ECFPs. (**b**) Plot of dataset P28335, when applying RDKit descriptors. The datasets correspond to those of Figure 3a and Figure 4a, respectively.

The fact that both UMs performed comparably gave rise to the question whether they were correlated. Examples of the SDEP vs. HVL correlation plots for two datasets are visualized in Figure 5. The datasets correspond to the examples in Figure 3a and Figure 4a. In the first case, P16581 using ECFPs, HVLs outperformed the SDEP. The dissimilarity of the two UMs in this case was also indicated by a rather low Pearson correlation coefficient (*r*) of 0.58. In the second case, P28335 using RDKit descriptors, both UMs performed alike. The values of both UMs for each object were also more correlated, resulting in an *r* of 0.78. Overall, values of *r* ranged between 0.39 (P17252 with RDKit descriptors) and 0.86 (FREESOLV with RDKit descriptors). Scatter plots and *r*s for all cases are provided in Tables S7 and S8 in the Supporting Information.

### 2.3. Decline in MSE When Omitting the Least Certain Predictions

**Figure 6:**
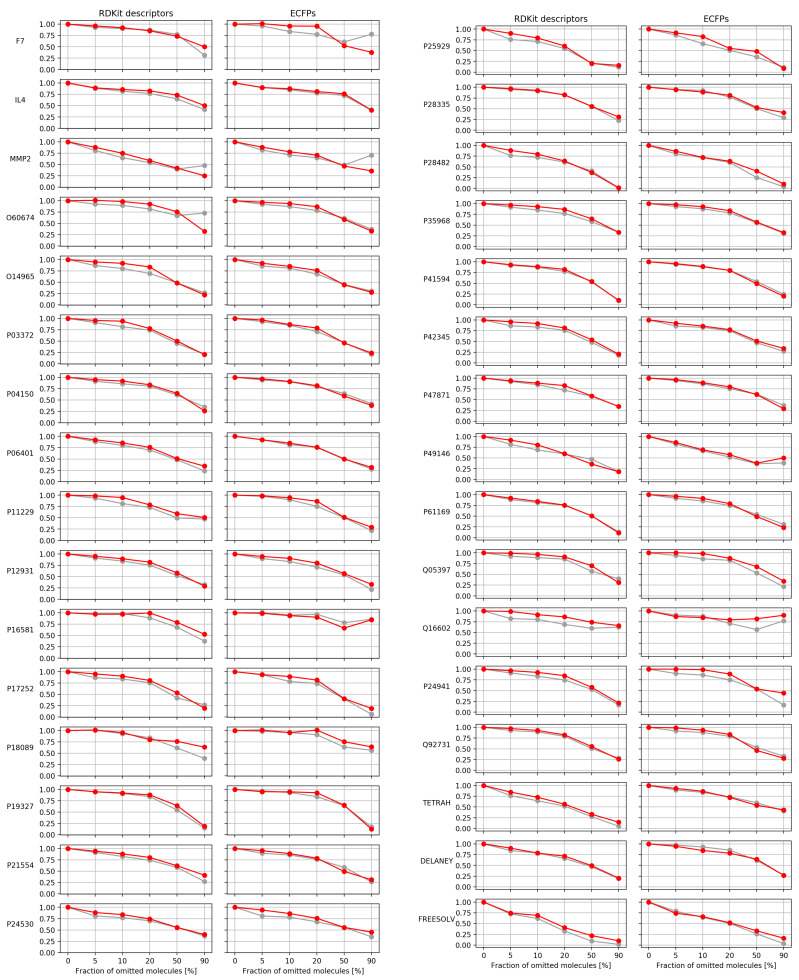
MSEs for different fractions of omitted predictions for all datasets. The label of the corresponding dataset of each plot is denoted left to the *y*-axis. MSEs when removing predictions according to the SDEP are shown in gray. MSEs obtained by removing predictions using HVLs are depicted in red. For each evaluation, the six connected points correspond to the MSE when leaving out the least certain 0%, 5%, 10%, 20%, 50%, and 90%, as denoted by the *x*-axis. All values within each evaluation were divided by the total MSE involving all predictions ($MSE_{0}$) to aid visual comparison. The numbers on each *y*-axis are, therefore, fractions of $MSE_{0}$.

The MSEs for leaving out the least certain fractions of different sizes are visualized in Figure 6. For most cases, the MSE constantly shrank when removing more unreliable predictions according to both UMs. In some evaluations, the MSE rose from one step to the next. Such reversions could mostly be observed from $MSE_{50}$ to $MSE_{90}$ (i.e., in cases least relevant for practitioners); overall, in seven evaluations. Four of those incidents solely arose from the usage of the SDEP. The corresponding evaluations belonged to the datasets MMP2 and O60674 when using RDKit descriptors, and F7 and MMP2 when using ECFPs. In the other three cases, the effect occurred for HVLs and the SDEP alike. They corresponded to P16581, P49146, and Q05397, all featurized by ECFPs. Rises in MSE from one fraction to the next could also be observed for other steps. Exemplarily, $MSE_{20}$ was larger than $MSE_{10}$ for P18089 when ECFPs and HVLs were applied. Apart from these exceptions, the trend already observed for the $AUCO_{50}$ continued beyond the 50% area.

The step from $MSE_{0}$ to $MSE_{5}$ represents the ability to detect the least accurate predictions. In some cases, the $MSE_{5}$ was larger than the $MSE_{0}$, implying that the UM was misleading for these fractions and eliminated accurate predictions. Examples thereof comprise the evaluation with HVLs of dataset P18089 for RDKit descriptors, or that of F7 in combination with ECFPs. This behavior was found exclusively for evaluations involving HVLs.

**Figure 7:**
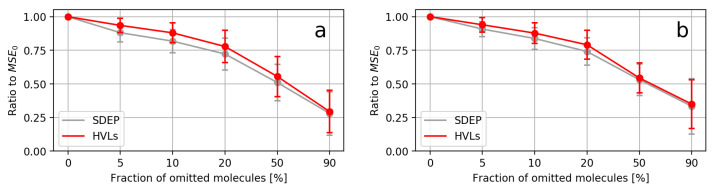
Average MSEs for each fraction across all datasets. The vertical bars at each point indicate standard deviations. For comparability, all MSEs were divided by the $MSE_{0}$ before averaging. (**a**) Plot for evaluations using RDKit descriptors. (**b**) Plot for evaluations using ECFPs.

Overall, most SDEP MSEs were below their corresponding HVL MSEs. All $MSE_{50}$s and all $MSE_{90}$s, independent of the dataset, descriptor, or UM, were below their $MSE_{0}$s. The average MSEs at each fraction across all datasets for both descriptors is summarized in Figure 7. It can be seen that the standard deviations between the measures overlapped for all average MSEs. With the exception of the average $MSE_{5}$ in the case of RDKit descriptors, the average MSEs of HVLs lied within the standard deviations of the corresponding SDEP MSEs and vice versa. Despite some increases from one fraction to the next, Figure 7 reveals that, on average, both UMs caused a decline in MSE when applied to remove unreliable predictions.

An overview of all $AUCO_{50}$s and MSEs of the specified fractions is provided in Tables S9–S12 in the Supporting Information.

## 3. Discussion

In all cases, the MSE decreased when removing uncertain predictions according to each measure. For a random measure with no correlation to prediction uncertainty, the MSE is expected to remain constant on average. Therefore, both UMs were considered to be effective in the detection of uncertain predictions. For the datasets and the evaluation strategy at hand, the SDEP outperformed HVLs in most cases, although their performances did turn out to be comparable. Despite the predictive quality of the examined model or its degree of overfitting, the decline in error could be considered similar for both UMs. Especially in cases where both measures showed similar performances, variations in the selection of train and test splits might change the ranking. The results would also differ when the standard deviation threshold was tuned as a hyperparameter. In this case, it also had to be decided how to tune the threshold, i.e., which objective function to use for this optimization step. AUCO would be a natural choice, but the MSE for a given fraction of the predictions could also be used.

To a certain degree, the fraction of HVLs and the SDEP were correlated in all cases, which was expected, since both measures were effectively able to eliminate uncertain predictions. The absence of a correlation could only be expected from a random measure unrelated to prediction errors. Furthermore, HVLs detect local variations, i.e., output variability in similar regions of the independent variables. The SDEP is based on the variation of the predictions across the trees. In many cases, local variation in the leaves can induce mean shifts of the predictions. Thus, in these cases, both measures can yield similar uncertainties. Yet, cases are conceivable where homogeneous leaves in different trees can still disagree in their predictions, which can go unnoticed by HVLs, but can be detected by the SDEP. This might be the reason why the SDEP performed slightly better. This line of reasoning showed an advantage of the SDEP and underlined its value as the default UM in regression RFs.

Cases of strong correlation further indicated the similarity of both UMs. In cases of weaker correlation, both UMs could be combined in expectation to benefit from synergistic effects. A consensus measure could be more effective than the two UMs individually. However, the aggregation of UMs was beyond the scope of this study.

By removing more and more unreliable predictions, each remaining MSE should be smaller than the previous MSE. For almost all observed cases and the choice of fraction size at each step, such a relation could be observed. Occasionally, the removal of larger fractions resulted in a larger MSE, at least between individual steps. Of those cases where the $MSE_{90}$ was larger than the $MSE_{50}$, three were also among models of least predictive performance. MMP2 and O60674, when using RDKit descriptors, and Q05397 when using ECFPs, were within the top four worst predictive models of each descriptor. On the one hand, it seems plausible that the success of a UM can be linked to the predictive quality of the model. It appears odd that ensemble members that highly disagree can be correct in their aggregated prediction. On the other hand, ensemble predictors benefit from the diversity of their members [23]. The random variable selection in RFs is actually a feature to induce member diversity since, depending on the dataset, a diverse set of predictions can still lead to an accurate single prediction. Exemplarily, Q16602, when applying ECFPs, also exhibited a rise in MSE from 50% to 90%, but was actually among the best performing models. It is, therefore, hardly generalizable to estimate the UM performance from the predictive performance.

Averaging over all results provided a more general view in contrast to considering the outcome of only some individual datasets. On average, the general decline in MSE and the observation that SDEP MSEs were below the MSESs of HVLs became clearer. Furthermore, by also including the standard deviation of the fraction-specific MSEs across all datasets, the overlap between both performances became visible, despite the fact that the SDEP outperformed HVLs. Variation between MSEs was more distinct when removing larger fractions, observable from the larger standard deviations for the $MSE_{20}$s, $MSE_{50}$s, and $MSE_{90}$s in contrast to those of the $MSE_{5}$s and $MSE_{10}$s. In conclusion, the reduction in MSE was similar for the most uncertain predictions, while the efficiency in error reduction in more certain predictions was rather case-dependent.

In general, a UM is not expected to reproduce the prediction error, but to estimate the confidence of the model for a particular new object. A metric that is able to actually estimate the error implies that the model probably left out usable information, i.e., was underfitted. The concept of predictive uncertainties is related to that of error bars (i.e., of confidence or prediction intervals), where larger error bars do not necessarily indicate larger errors, but a higher chance to find largely varying predictions.

Finally, predictive uncertainties depend on measurement errors in the training data. Models that yield accurate predictions based on experimental measurements are not necessarily able to accurately predict error bars. This phenomenon was pointed out by Wood et al. in a study about QSAR model quality, exemplary for a dataset of experimentally acquired lipophilicities [24]. For FREESOLV, the error reduction (relative to the total MSE) was most effective among all datasets, regardless of which descriptor or UM was used. Apart from the hydration free energies taken from the literature, the outputs for FREESOLV were simulated using methods of molecular dynamics. Thus, they were calculated instead of measured and, therefore, of a different nature, which might explain the extraordinary success of both UMs.

## 4. Materials and Methods

### 4.1. Data Acquisition

Overall, 32 datasets were examined. Of those datasets, 29 were activities compiled for a QSAR benchmarking study by Cortes-Ciriano. The number of compounds ranges from 137 to 4662 per set. The labels used in this article to refer to each activity dataset were identical to those in the previous study. To show the applicability of HVLs beyond the scope of the QSAR context, three additional datasets were added, which were not related to target activity. A set of toxicity values against *Tetrahymena pyriformis*, measured as inhibitory growth concentration, containing 1571 molecules, and was labeled TETRAH [25]. The ESOL dataset, referred to by Delaney, has 1144 entries of molecules and their aqueous solubility [26]. The dataset FREESOLV comprises 643 compounds and their hydration free energies, either taken from the literature or obtained by molecular dynamics simulations [27]. A comprehensive summary of all datasets can be found in Tables S13 and S14 in the Supporting Information.

### 4.2. Data Preparation

Initially, all compounds were retrieved as SMILES. The filtering steps that Cortes-Ciriano describes in their study were also applied to the activity datasets here. The implementation of the filtering mechanism is included in Figure S2 in the Supporting Information. The SMILES were converted to canonical SMILES using RDKit [28], followed by two descriptor calculations using the MoleculeNet framework [29], resulting in 64 cases for evaluation. The first descriptor was the extended connectivity fingerprints (ECFPs) [30] with an atom neighbor radius of three, hashed to bit vectors of a length of 2048. The second one was a collection of 200 physicochemical and fragment-based molecular properties from RDKit, called RDKit descriptors. IC_50_ values in the activity datasets were converted to pIC_50_ values by multiplying the decadic logarithm of the IC_50_ values by −1. The columns remained unscaled as RFs do not require variable standardization.

### 4.3. Machine Learning Setup

A single 10-fold cross-validation was performed to obtain test predictions for all molecules. The RF implementation of choice was the RandomForestRegressor from scikitlearn [31], with a fixed number of 500 ensemble members and a minimum of two samples per leaf. This ensured that every leaf had technically enough values to calculate a standard deviation. No hyperparameter tuning of RF took place, as the aim of this work was to assess UMs and not to improve predictive performance. The lack of hyperparameter tuning decreases overfitting and yields more comparable models. Variables that contained identical values, i.e., carried no information, were dropped at every iteration right before the current model was fitted. Due to the 10-fold approach, the output of every molecule was computed nine times when in a training split and one time when in a test split. To obtain single values for all molecules, the nine train values per molecule were averaged before any performance measures were computed. Similarly, the HVL occurrences for all train values were summed up and divided by the sum of all ensemble members in all models they could occur in (500 ∗ 9 = 4500).

### 4.4. Predictive Quality

Both UMs were presented in a competitive approach. The applicability of HVLs was revealed by demonstrating how well they performed compared to the state-of-the-art SDEP. However, such entirely relative comparisons did not account for cases in which the SDEP did not perform well in the first place. Therefore, it was reasonable to inspect the predictive performance first to obtain an impression of how erroneous the unreliable predictions actually were.

Two metrics were applied to evaluate predictive performances. The mean squared error (MSE) is an established metric to assess the error (absolute measure) for a set of predictions and corresponding observed outputs:

$$MSE=\frac{\sum_{i=1}^{n}{\left(y_{i}-{\hat{y}}_{i}\right)}^{2}}{n}.$$

Here, *n* refers to the number of data points in the test set, and $y_{i}$ and ${\hat{y}}_{i}$ are the observed values and their predictions, respectively. In contrast to the MSE, $R^{2}$ is a relative measure that compares the quality of the predictions to the single-valued mean predictor [32]:

$$R^{2}=1-\frac{\sum_{i=1}^{n}{\left(y_{i}-{\hat{y}}_{i}\right)}^{2}}{\sum_{i=1}^{n}(\bar{y}-y_{i}{)}^{2}}.$$

Various definitions of $R^{2}$ exist [33]. In case train data and test data are distinct sets, some definitions exclusively consider the average of the train outputs or test outputs as $\bar{y}$ [34]. Since the current implementation involved the whole dataset as train and test set alike (although keeping train and test distinct for every split), the usage at hand corresponded to the general definition of $R^{2}$ here, i.e., $\bar{y}$ included all outputs of a dataset.

### 4.5. Uncertainty Assessment

The implementation from scikit-learn does not simply calculate the average of all sample outputs in a leaf. From those outputs, some are selected and weighted unequally to further improve the predictive performance of the tree. Therefore, the prediction determined by a decision tree in the RandomForestRegressor does not correspond to the plain mean. The detection of HVLs was still implemented as explained, since the idea was to capture the output fluctuation in the subspace defined by the leaf. Taking the outputs obtained by the additional improvement step of scikit-learn would underestimate the leaf variance. The threshold to define HVLs was set to half the standard deviation in *y* of the current dataset. e.g., the outputs of TETRAH had a standard deviation of about 1.03, so the threshold for this dataset was approximately 0.515. This procedure led to reasonable results and was based on preliminary experiments.

Comparing the SDEP to HVLs was performed by assessing their abilities to eliminate uncertain predictions. In other words, given as a set of predictions, how well does the overall error decrease when removing the worst predictions according to some measure? A good UM would be able to detect the worst predictions without knowing their errors. Thus, removing those predictions should decrease the MSE, where a better UM would decrease the MSE further while removing the same amount of predictions. For both UMs, the predictions were sorted from least to most certain, i.e., the highest SDEP to the lowest SDEP and most HVLs to least HVLs, respectively. Additionally, the predictions were sorted by their absolute residual, descendingly, to emulate an ideal measure that perfectly scales with the error. All MSEs from leaving out 0% to 50% of the predictions according to each measure were computed object-wise and plotted for comparable MSE decrease plots, or confidence curves. Hereby, the MSE was re-calculated every time a single prediction was removed, where the $MSE_{0}$ contained all *n* predictions of the dataset, the next point on the confidence curve contained n−1 predictions, etc. The ideal curve, where the predictions were perfectly sorted (by their residual) would, therefore, decrease sharply and smoothly, while the curves where the residuals were sorted by a UM would practically never follow this ideal shape. The areas that the curves of both UMs enclosed with the ideal measure curve were compared to assess the difference in their uncertainty measurement performance. Scalia et al. refer to this metric by area under the confidence–oracle error (AUCO) [23]. The AUCO of 50% data coverage would be referred to by $AUCO_{50}$. A smaller $AUCO_{50}$ implies that the curve of the measure was closer to the ideal curve; thus, removing erroneous predictions more efficiently. Covering the full area, i.e., up to 100%, leads to unreliable results due to the division by the number of remaining objects which becomes smaller and smaller.

To inspect the detection of unreliable predictions beyond 50%, but also to compare smaller fractions, the remaining MSEs at 5%, 10%, 20%, 50%, and 90% prediction removal were picked, resulting in $MSE_{5}$, $MSE_{10}$, $MSE_{20}$, $MSE_{50}$, and $MSE_{90}$. These resulting MSEs were compared between the two UMs.

## 5. Conclusions

In this work, HVLs were evaluated as a specific UM for RFs. Their principle was explained and their performance estimated on a diverse collection of datasets. It could be demonstrated that the concept of HVLs was applicable to assess predictive uncertainties and comparable in performance to the default SDEP. Especially due to their similar efficiency, the large-scale evaluations were required to reasonably compare the two UMs. In other words, a smaller study could misleadingly suggest that HVLs prevail over the SDEP, e.g., by only involving the non-bioactivity datasets and ECFPs.

An additional variation of the HVL implementation could improve the detection of unreliable predictions. A continuous definition of the UM could increase its performance. Instead of a hard cutoff, a continuous estimator, e.g., the average leaf variance, could reproduce the output fluctuation more distinctly. With a continuous estimator, there is no need for setting the cutoff value, which is an additional hyperparameter. The immediate disadvantage of the continuous version of the measure is that it can become infinitely large and is not normalized.

In contrast to the SDEP, which can be used for all ensemble regressors, HVLs represent a measure tailored to RF. The perception that areas of unusual deviations are harder to model is intuitive and makes HVLs easy to infer from the training data. However, while HVLs reliably detect local variations (in the sense of heterogeneous outputs for similar molecules), the SDEP also considers the impact of local variations on the predictions, which might be the reason why the SDEP was slightly more effective.

## Figures and Tables

**Table 1:** Train performances and test performances for every dataset in combination with both descriptors. All calculations were performed at full floating-point precision, and the table was rounded afterwards. For the descriptor that achieved the better test performance on a particular dataset, Test MSE and Test $R^{2}$ are underlined and in bold. The median of each column is presented in the last row.

	RDKit Descriptors				ECFPs			
	**Train**		**Test**		**Train**		**Test**	
**Dataset**	***MSE***	***R*** **^2^**	***MSE***	***R*** **^2^**	***MSE***	***R*** **^2^**	***MSE***	***R*** **^2^**
F7	0.049	0.948	**0.277**	**0.705**	0.077	0.918	0.303	0.676
IL4	0.027	0.912	0.146	0.520	0.036	0.883	**0.133**	**0.563**
MMP2	0.084	0.905	0.465	0.474	0.102	0.885	**0.389**	**0.560**
O60674	0.133	0.909	0.742	0.495	0.139	0.905	**0.597**	**0.594**
O14965	0.106	0.938	0.610	0.642	0.104	0.939	**0.449**	**0.736**
P03372	0.097	0.945	**0.489**	**0.720**	0.120	0.931	0.497	0.715
P04150	0.085	0.936	0.444	0.664	0.110	0.917	0.445	0.664
P06401	0.070	0.947	0.399	0.696	0.089	0.932	**0.373**	**0.715**
P11229	0.087	0.951	**0.500**	**0.717**	0.129	0.927	0.522	0.705
P12931	0.100	0.947	0.546	0.712	0.113	0.940	**0.478**	**0.747**
P16581	0.108	0.937	0.620	0.640	0.140	0.919	**0.539**	**0.688**
P17252	0.109	0.928	0.539	0.642	0.125	0.917	**0.495**	**0.671**
P18089	0.103	0.932	**0.606**	**0.598**	0.122	0.919	0.640	0.575
P19327	0.096	0.924	0.565	0.553	0.107	0.915	**0.481**	**0.620**
P21554	0.095	0.955	0.556	0.736	0.115	0.945	**0.513**	**0.757**
P24530	0.068	0.958	0.368	0.770	0.077	0.952	**0.306**	**0.809**
P25929	0.081	0.955	0.473	0.739	0.091	0.950	**0.386**	**0.787**
P28335	0.088	0.923	0.519	0.541	0.109	0.904	**0.499**	**0.560**
P28482	0.074	0.915	0.404	0.532	0.077	0.911	**0.393**	**0.546**
P35968	0.109	0.934	0.648	0.608	0.119	0.928	**0.544**	**0.671**
P41594	0.113	0.910	0.664	0.473	0.133	0.894	**0.561**	**0.555**
P42345	0.095	0.963	0.534	0.790	0.105	0.959	**0.450**	**0.823**
P47871	0.079	0.929	0.451	0.591	0.097	0.912	**0.421**	**0.619**
P49146	0.071	0.954	**0.399**	**0.741**	0.096	0.938	0.423	0.726
P61169	0.087	0.910	0.498	0.486	0.100	0.897	**0.417**	**0.570**
Q05397	0.084	0.919	0.484	0.534	0.109	0.895	**0.471**	**0.546**
Q16602	0.073	0.968	0.439	0.808	0.100	0.956	**0.416**	**0.818**
P24941	0.115	0.940	0.661	0.655	0.136	0.929	**0.581**	**0.697**
Q92731	0.097	0.926	**0.496**	**0.619**	0.127	0.902	0.516	0.604
TETRAH	0.036	0.967	**0.190**	**0.822**	0.082	0.923	0.291	0.727
DELANEY	0.073	0.983	**0.413**	**0.906**	0.349	0.921	1.278	0.709
FREESOLV	0.281	0.981	**1.539**	**0.896**	1.351	0.909	4.516	0.694
Median	0.088	0.938	0.497	0.649	0.109	0.919	**0.474**	**0.682**

## Data Availability

Code is contained in the Supplementary Materials, models and predictions are available on request from the corresponding author.

## Supplementary Materials

The following are available online in the Supporting Information, Figure S1: scheme of a fitted regression tree with a high-variance leaf, Figure S2: Python code of the filtering function applied to the activity datasets, Tables S1 and S2: observed vs. predicted scatter plots, Tables S3 and S4: confidence curve plots for 50% data coverage, Tables S5 and S6: uncertainty vs. residual scatter plots, Tables S7 and S8: SDEP vs. HVLs scatter plots, Tables S9–S12: $AUCO_{50}$ and declining MSE, Table S13: all datasets used for evaluation, Table S14: distribution of the dependent variables, Table S15: Python packages with versions.

## Abbreviations

The following abbreviations are used in this manuscript:

AUCO	Area under the confidence–oracle error
HVL	High-variance leaf
MSE	Mean squared error
QSAR	Quantitative structure–activity relationship
RF	Random forest
SDEP	Standard deviation of ensemble predictions
UM	Uncertainty measure
