# Supplementary material for: Evaluating High-Variance Leaves as Uncertainty Measure for Random Forest Regression
Source: Molecules. 2021 Oct 28;26(21):6514. doi: 10.3390/molecules26216514 (PMC8588039; doi:10.3390/molecules26216514)
Supplement: Supplementary file 1 [file molecules-26-06514-s001.zip › supplementary_materials/supporting_information.pdf]

Supporting Information

Evaluating High-Variance Leaves as Uncertainty Measure for Random Forest Regression

Thomas-Martin Dutschmann[†] and Knut Baumann[†]

†*Institute for Medicinal and Pharmaceutical Chemistry, University of Technology Braunschweig, Beethovenstraße 55, 38106 Braunschweig, Germany*

E-mail: t.dutschmann@tu-braunschweig.de, k.baumann@tu-braunschweig.de

1 Illustration of a High-Variance Leaf

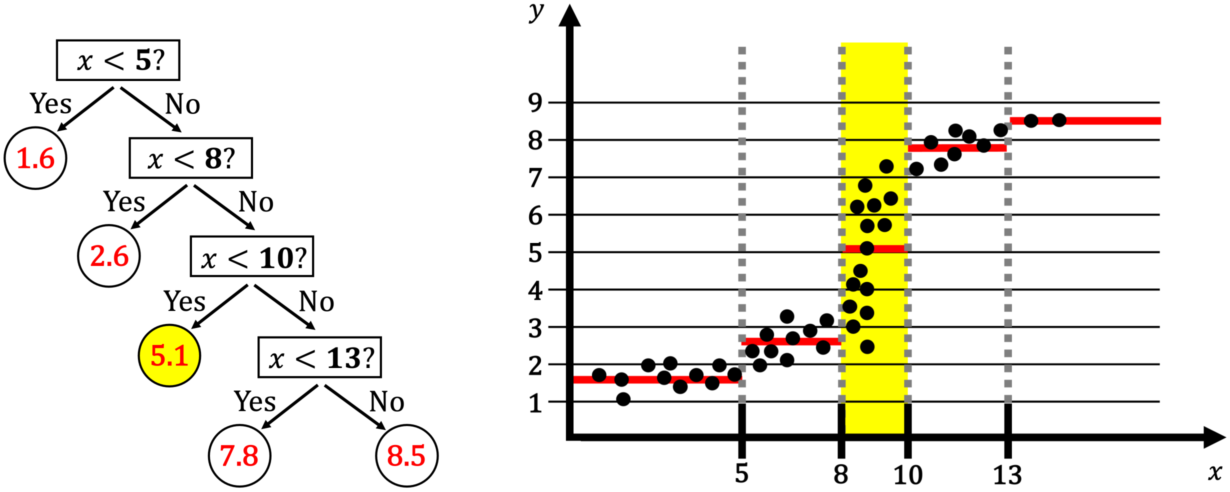

(a) (b)

Figure S1: Scheme of a fitted regression tree with a high-variance leaf. For the sake of simplicity, there is only one input variable x. (**a**) Visualization of the tree structure. Nodes with outgoing edges are depicted as boxes, leaves as circles. The decision rule is written in each node. The prediction of each leaf is colored in red. The leaf that is highlighted in yellow arises from an area in the data with particular high output variance. (**b**) The data that the tree was fitted to. The data points with their outputs in y are shown as black dots. The decision boundaries of the corresponding nodes in the tree that split the input variable are indicated by grey dashed lines. The mean values that the leaves predict are depicted as red bars. A comparatively small area in x with unusual high variance in y is highlighted in yellow.

2 Code of the Activity Data Set Filter Function

```python
import pandas as pd


def cortes_ciriano_filter(df):
    df_small = df[df['MOLECULE_TYPE'] == 'Small molecule']
    df_small_conf = df_small[df_small['CONFIDENCE_SCORE'] >= 8]
    df_small_conf_nm = df_small_conf[df_small_conf['STANDARD_UNITS'] == 'nM']
    df_small_conf_nm_mean = df_small_conf_nm[['STANDARD_VALUE', 'smiles']] \
        .groupby('smiles').mean().reset_index()
    return df_small_conf_nm_mean
```

Figure S2: Python-code of the filtering function applied to the activity data sets, following from the descriptions in the study of Cortes-Ciriano.

3 Observed vs. Predicted Scatter Plots (RDKit Descriptors)

Table S1.1: Observed vs. predicted scatter plots of each data set using RDKit descriptors 1–16.

F7

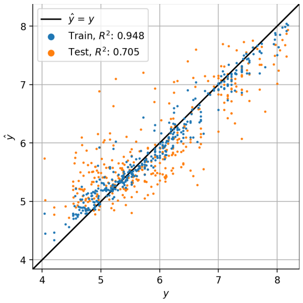

IL4

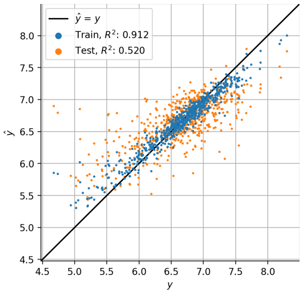

MMP2

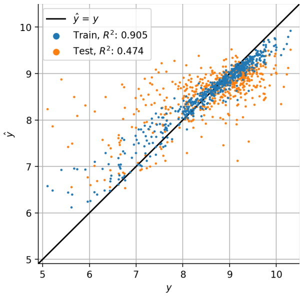

O60674

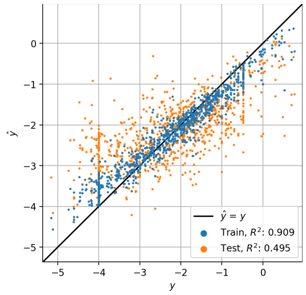

O14965

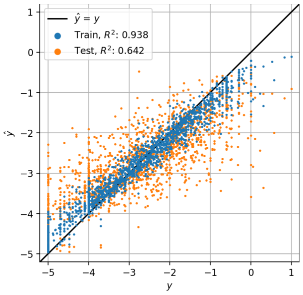

P03372

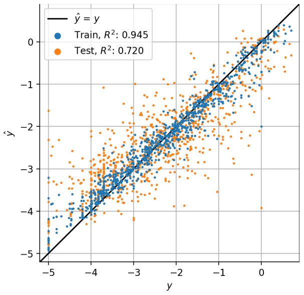

P04150

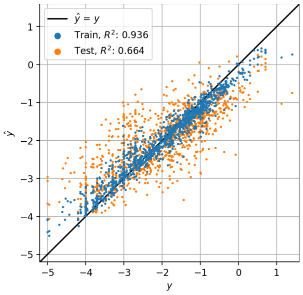

P06401

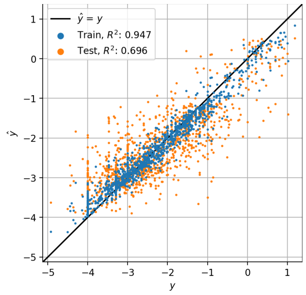

P11229

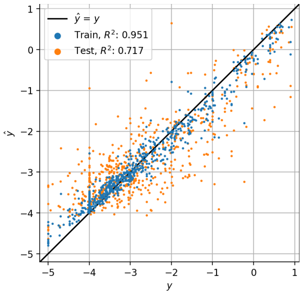

P12931

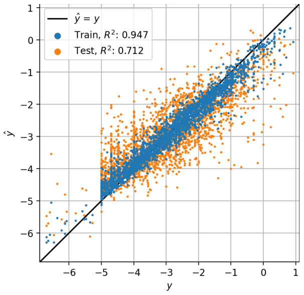

P16581

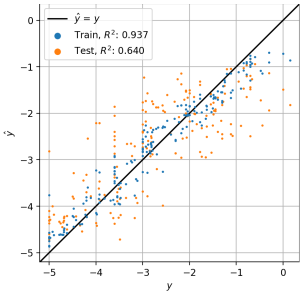

P17252

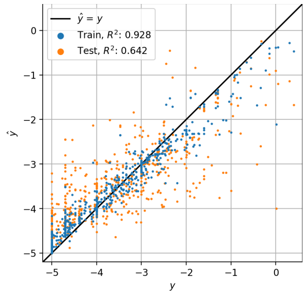

P18089

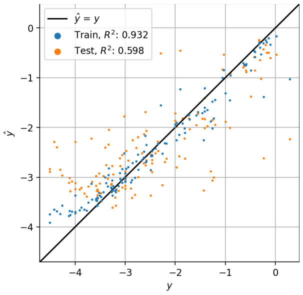

P19327

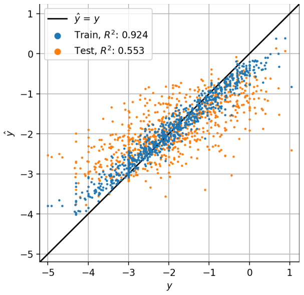

P21554

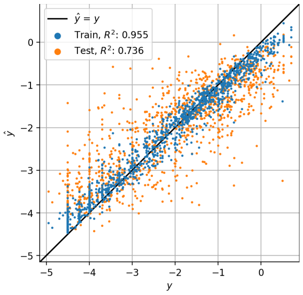

P24530

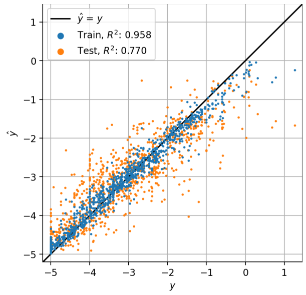

Table S1.2: Observed vs. predicted scatter plots of each data set using RDKit descriptors 17–32.

P25929

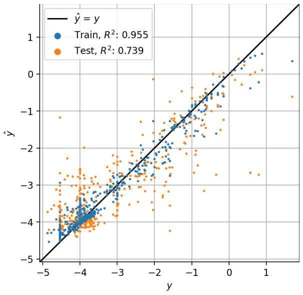

P28335

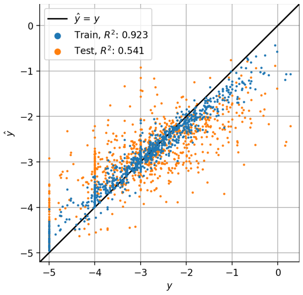

P28482

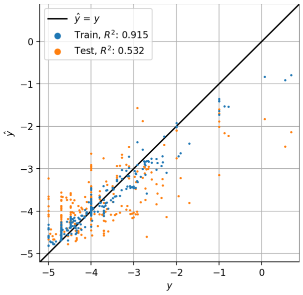

P35968

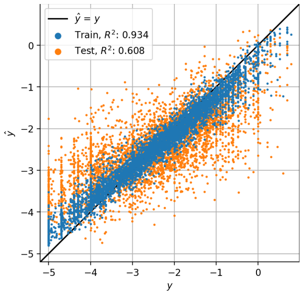

P41594

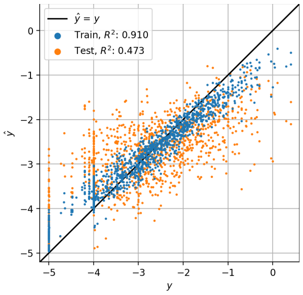

P42345

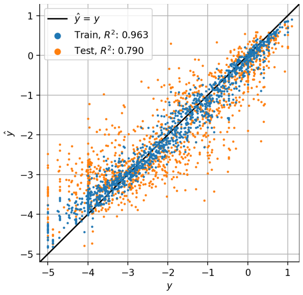

P47871

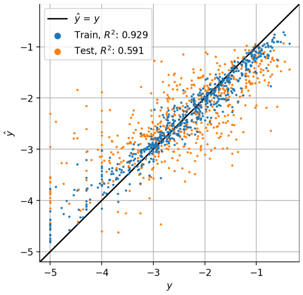

P49146

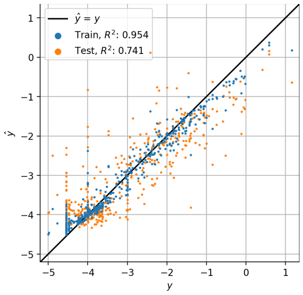

P61169

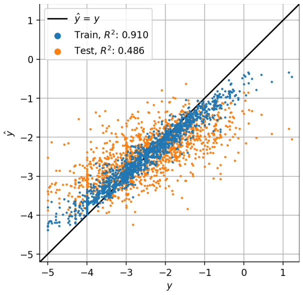

Q05397

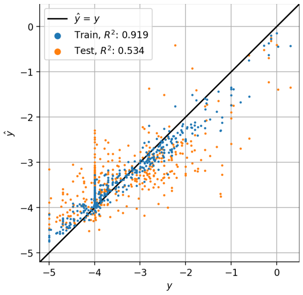

Q16602

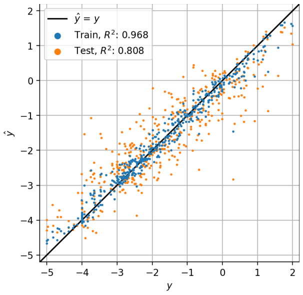

P24941

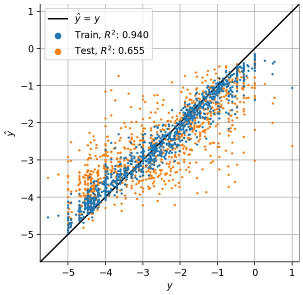

Q92731

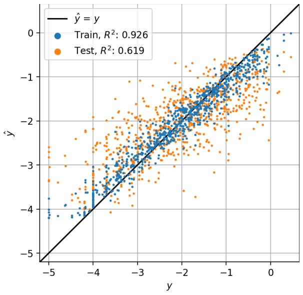

TETRAH

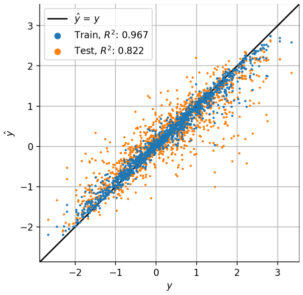

DELANEY

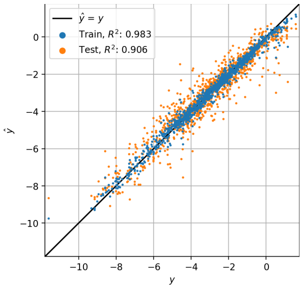

FREESOLV

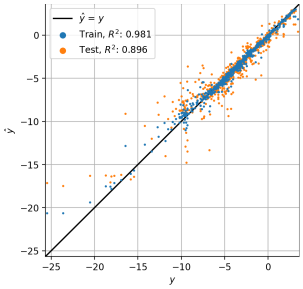

4 Observed vs. Predicted Scatter Plots (ECFPs)

Table S2.1: Observed vs. predicted scatter plots of each data set using ECFPs 1–16.

F7

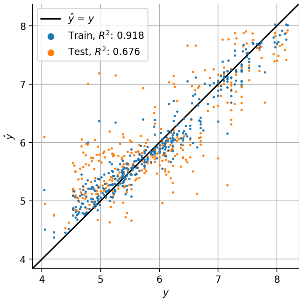

IL4

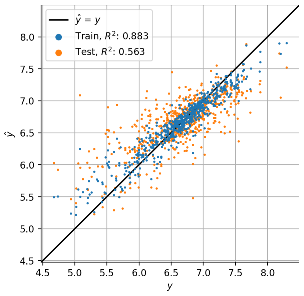

MMP2

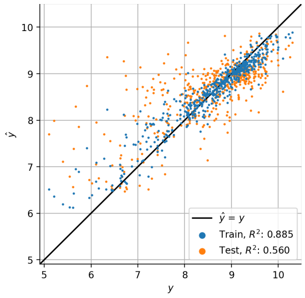

O60674

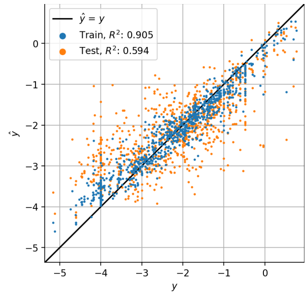

O14965

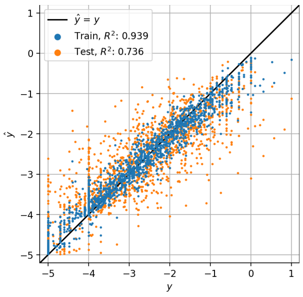

P03372

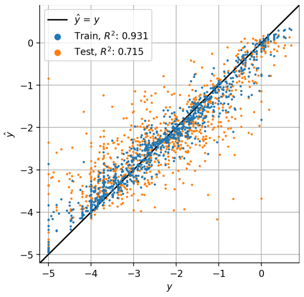

P04150

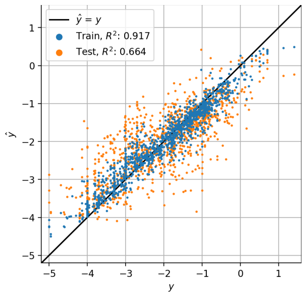

P06401

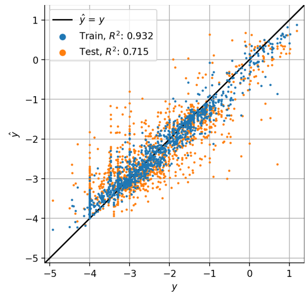

P11229

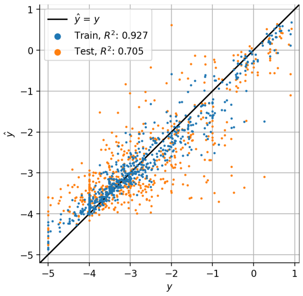

P12931

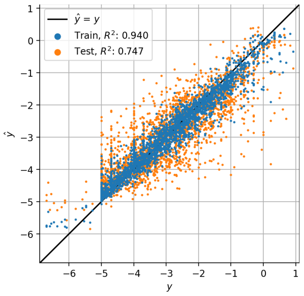

P16581

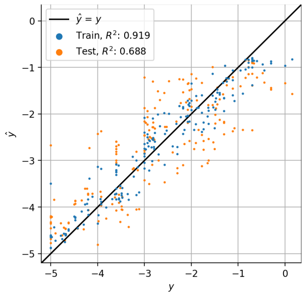

P17252

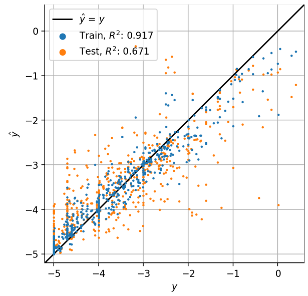

P18089

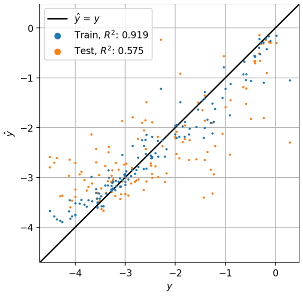

P19327

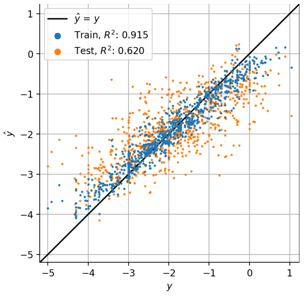

P21554

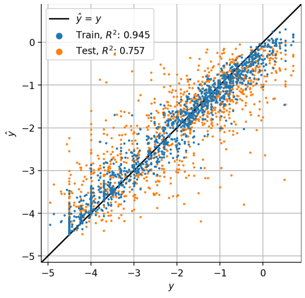

P24530

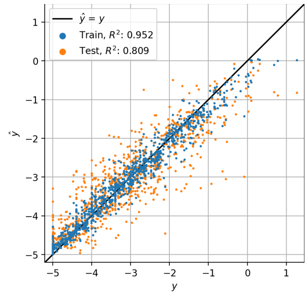

Table S2.2: Observed vs. predicted scatter plots of each data set using ECFPs 17–32.

P25929

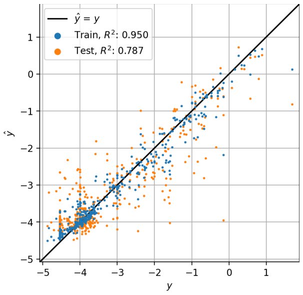

P28335

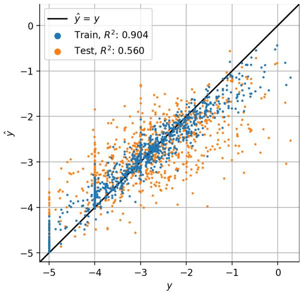

P28482

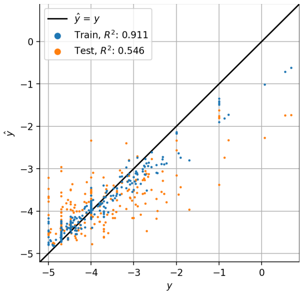

P35968

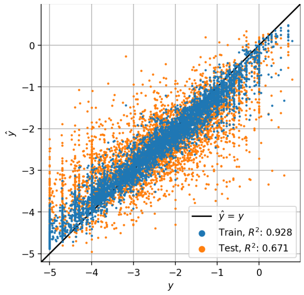

P41594

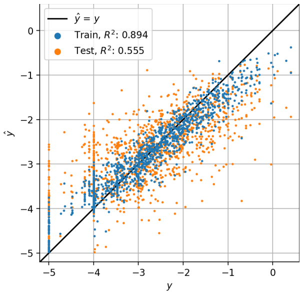

P42345

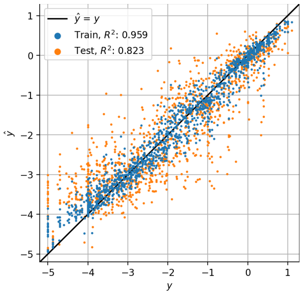

P47871

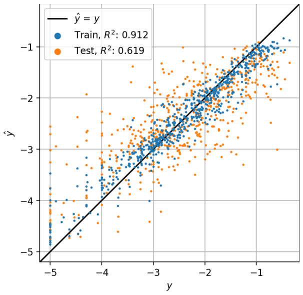

P49146

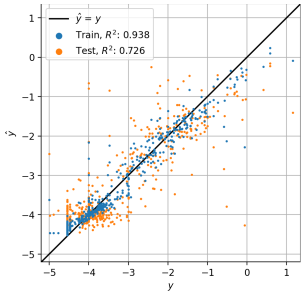

P61169

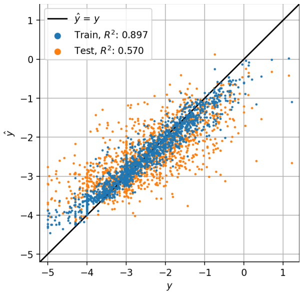

Q05397

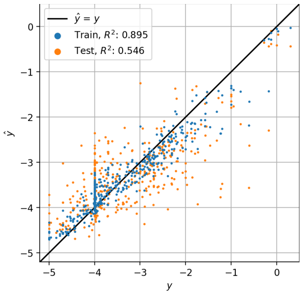

Q16602

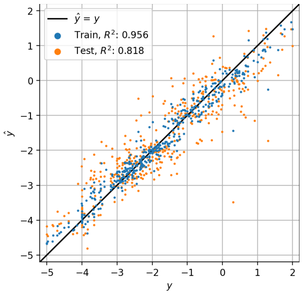

P24941

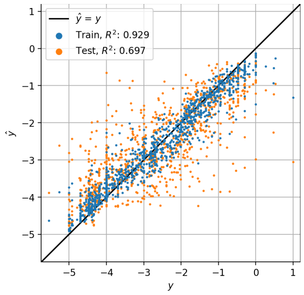

Q92731

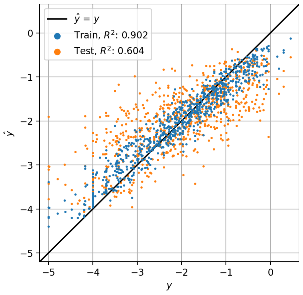

TETRAH

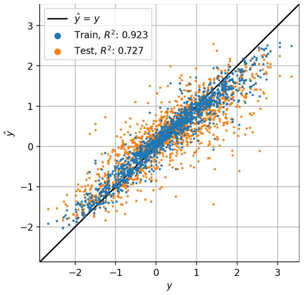

DELANEY

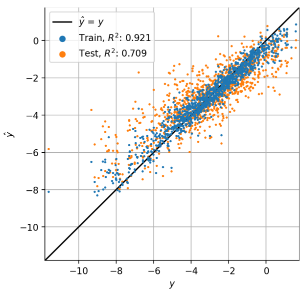

FREESOLV

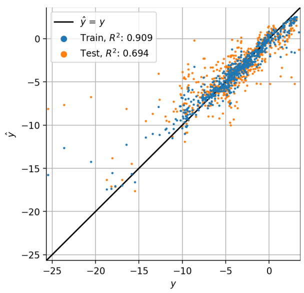

5 Confidence Curves (RDKit Descriptors)

Table S3.1: Confidence curve plots for 50 % data coverage of each data set using RDKit descriptors 1–28.

F7

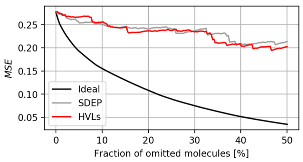

IL4

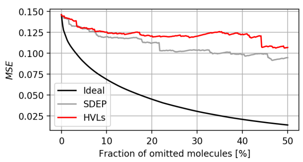

MMP2

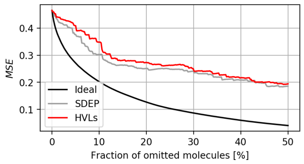

O60674

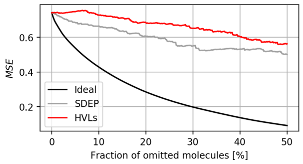

O14965

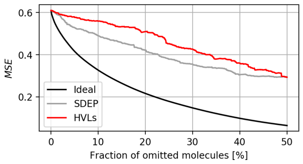

P03372

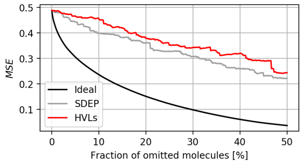

P04150

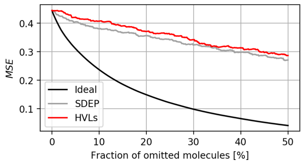

P06401

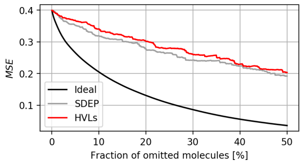

P11229

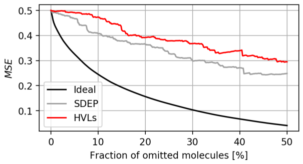

P12931

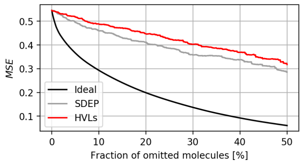

P16581

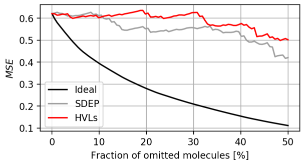

P17252

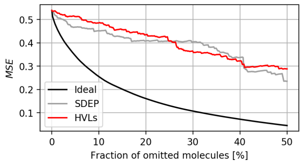

P18089

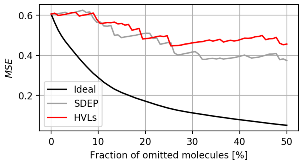

P19327

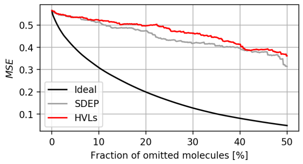

P21554

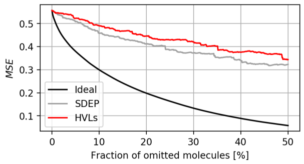

P24530

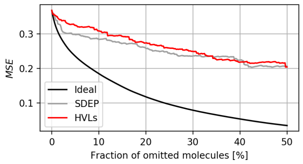

P25929

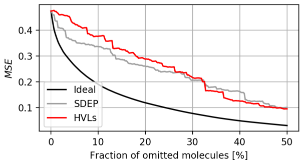

P28335

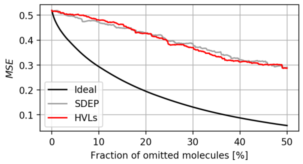

P28482

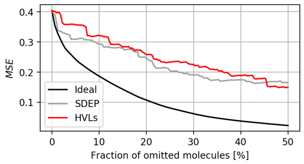

P35968

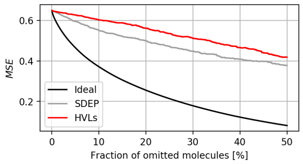

P41594

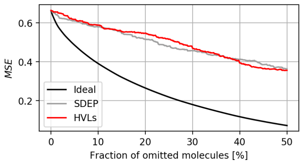

P42345

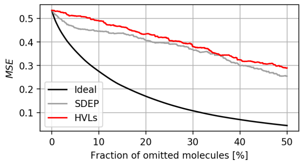

P47871

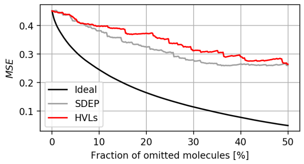

P49146

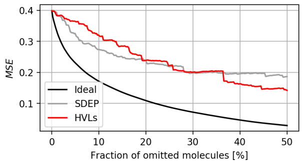

P61169

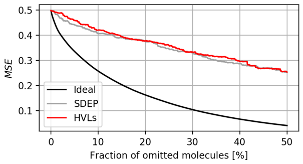

Q05397

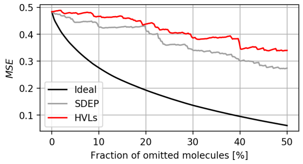

Q16602

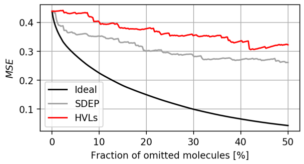

P24941

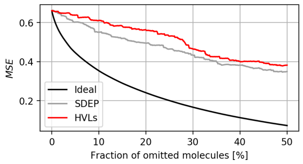

Table S3.2: Confidence curve plots for 50 % data coverage of each data set using RDKit descriptors 29–32.

Q92731	TETRAH	DELANEY	FREESOLV
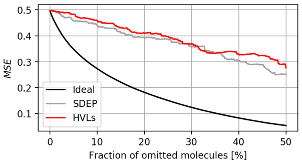	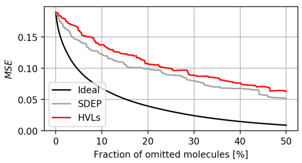	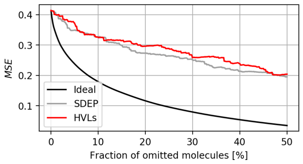	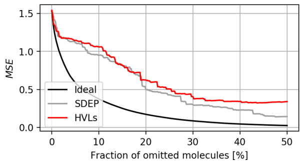

6 Confidence Curves (ECFPs)

Table S4.1: Confidence curve plots for 50 % data coverage of each data set using ECFPs 1–20.

F7	IL4	MMP2	O60674
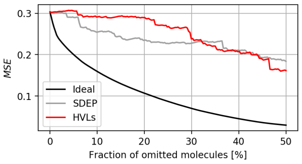	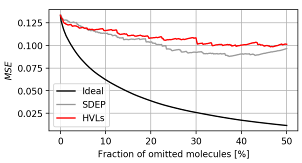	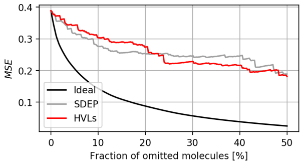	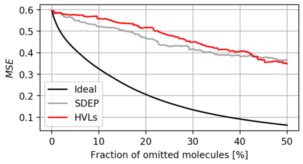
O14965	P03372	P04150	P06401
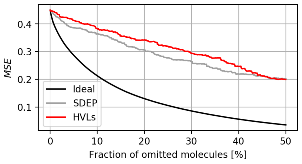	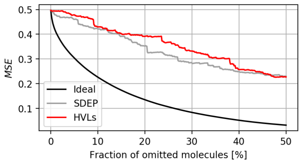	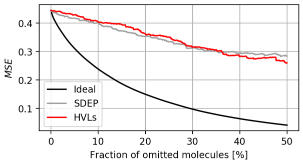	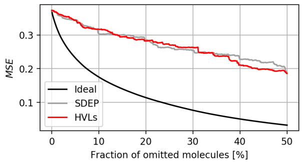
P11229	P12931	P16581	P17252
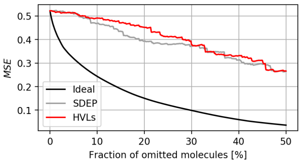	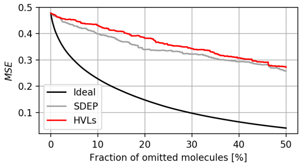	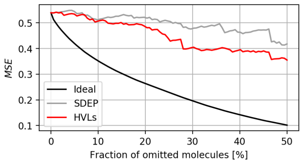	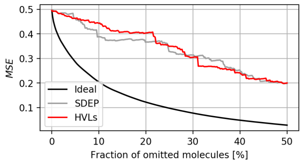
P18089	P19327	P21554	P24530
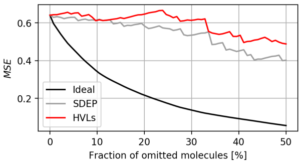	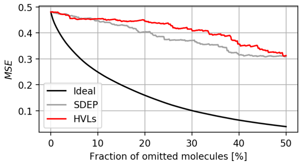	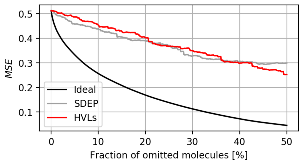	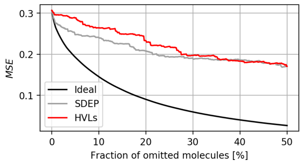
P25929	P28335	P28482	P35968
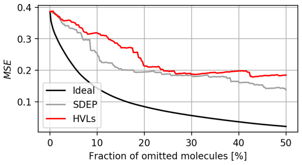	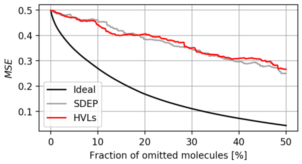	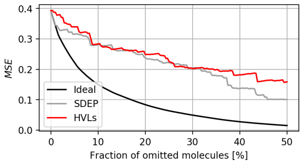	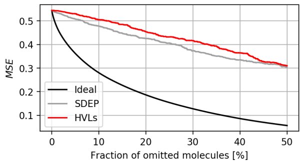

Table S4.2: Confidence curve plots for 50 % data coverage of each data set using ECFPs 21–32.

P41594

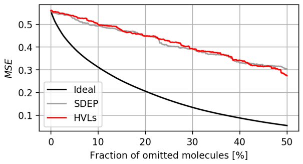

P42345

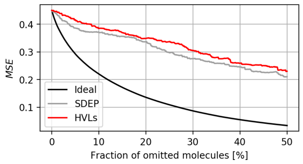

P47871

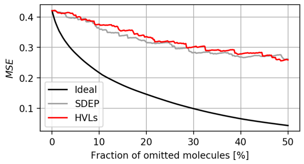

P49146

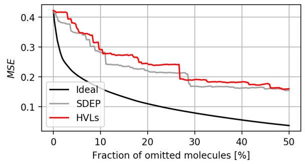

P61169

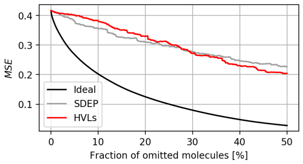

Q05397

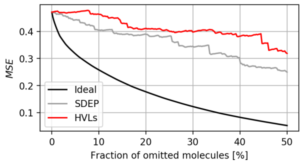

Q16602

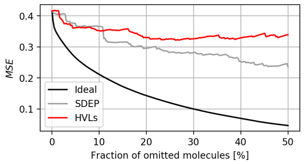

P24941

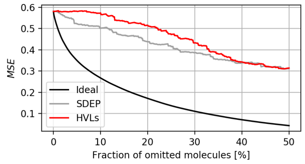

Q92731

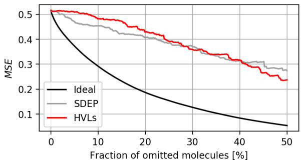

TETRAH

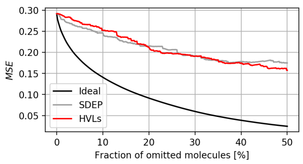

DELANEY

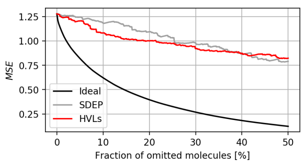

FREESOLV

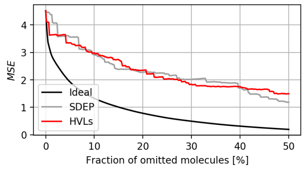

7 Uncertainty vs. Residual Scatter Plots (RDKit Descriptors)

Table S5.1: Uncertainty vs. residual scatter plots of each data set using RDKit descriptors 1–12.

F7

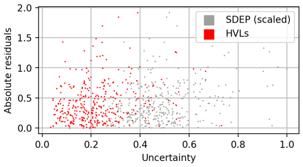

IL4

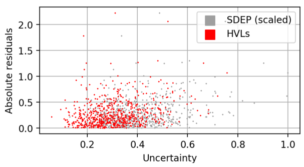

MMP2

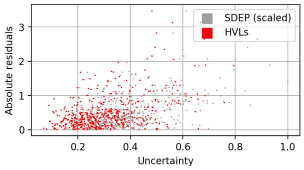

O60674

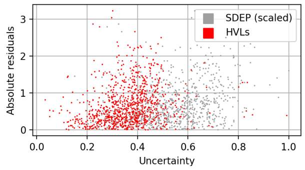

O14965

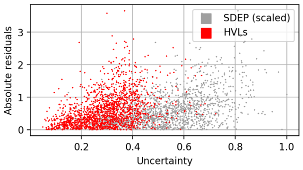

P03372

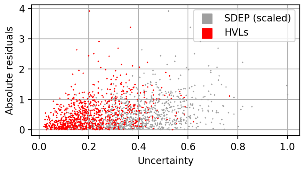

P04150

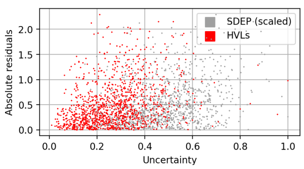

P06401

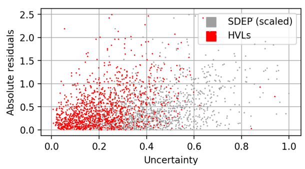

P11229

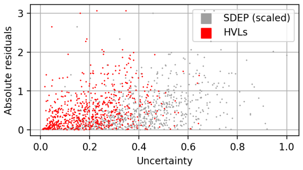

P12931

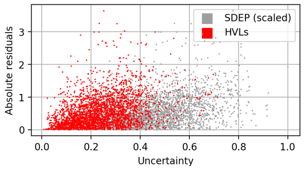

P16581

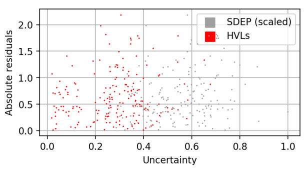

P17252

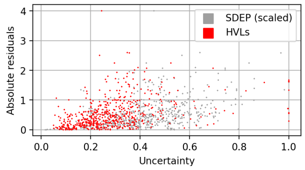

Table S5.2: Uncertainty vs. residual scatter plots of each data set using RDKit descriptors 13–32.

P18089

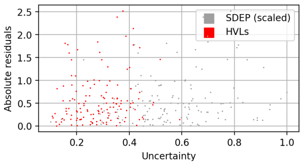

P19327

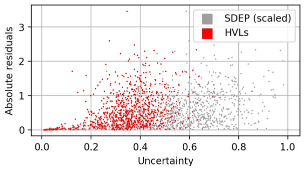

P21554

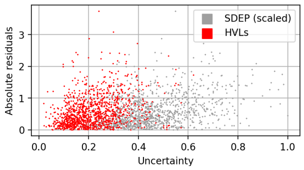

P24530

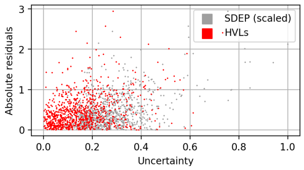

P25929

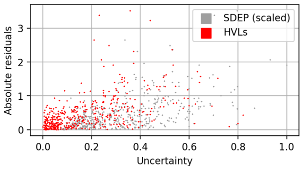

P28335

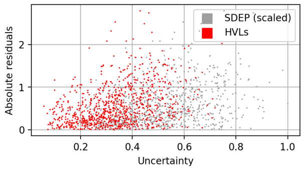

P28482

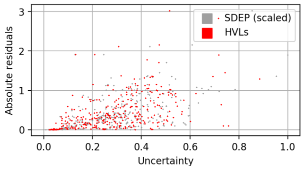

P35968

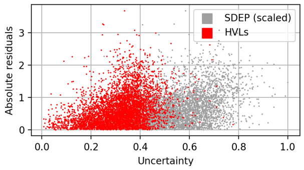

P41594

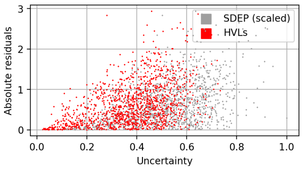

P42345

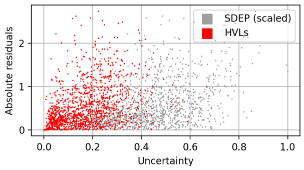

P47871

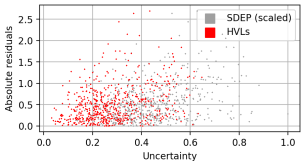

P49146

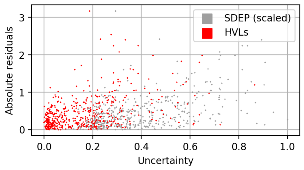

P61169

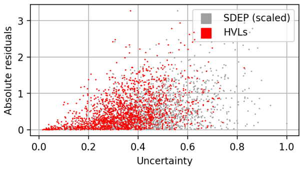

Q05397

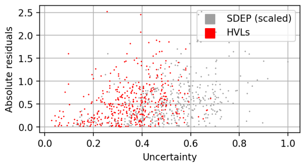

Q16602

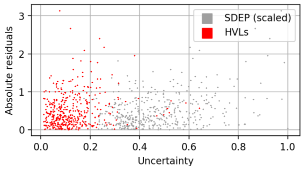

P24941

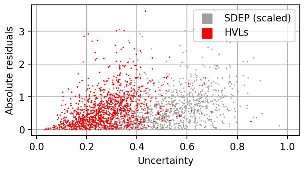

Q92731

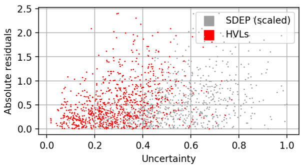

TETRAH

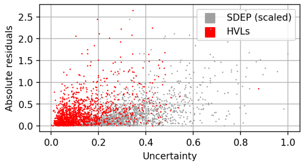

DELANEY

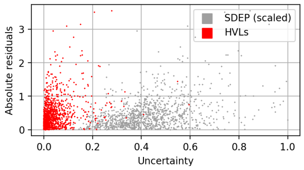

FREESOLV

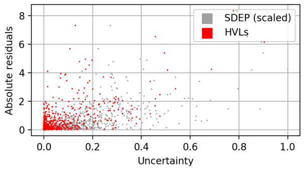

8 Uncertainty vs. Residual Scatter Plots (ECFPs)

Table S6.1: Uncertainty vs. residual scatter plots of each data set using ECFPs 1–4.

F7

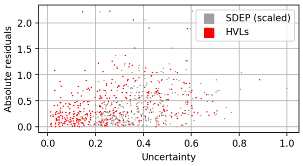

IL4

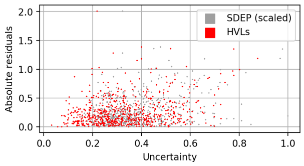

MMP2

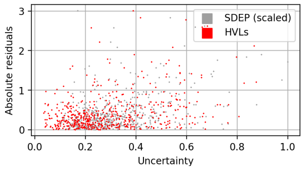

O60674

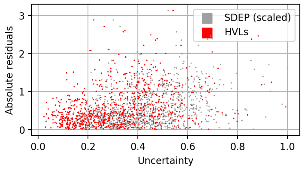

Table S6.2: Uncertainty vs. residual scatter plots of each data set using ECFPs 5–32.

O14965

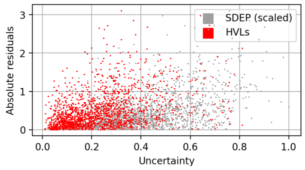

P03372

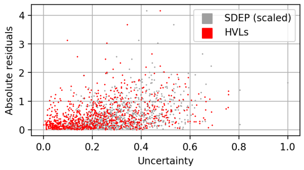

P04150

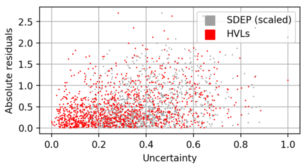

P06401

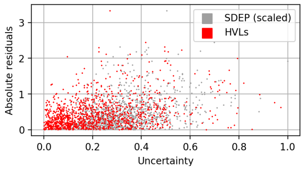

P11229

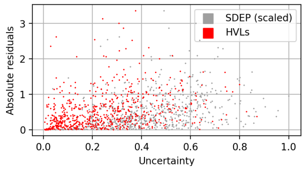

P12931

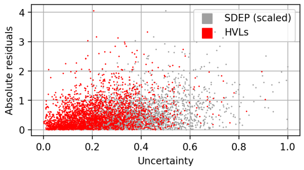

P16581

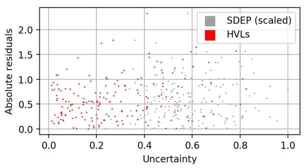

P17252

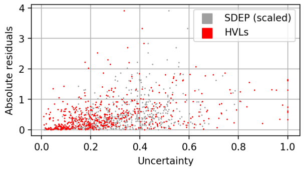

P18089

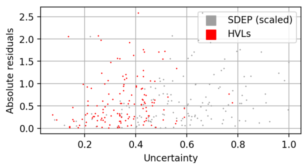

P19327

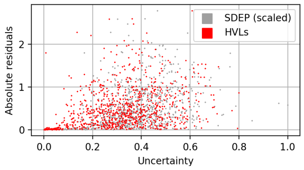

P21554

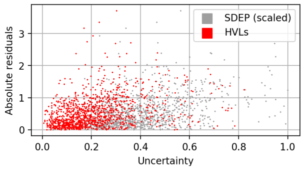

P24530

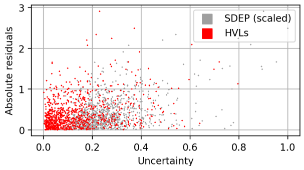

P25929

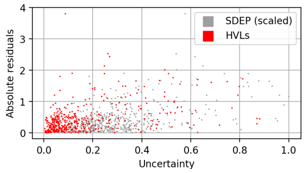

P28335

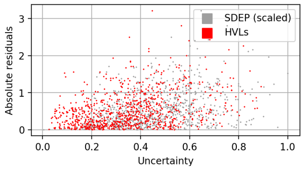

P28482

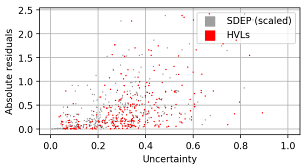

P35968

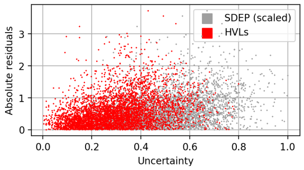

P41594

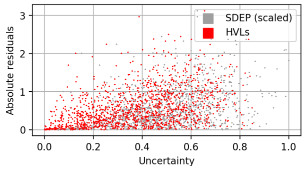

P42345

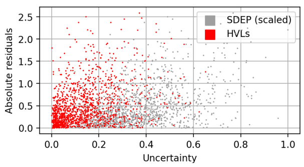

P47871

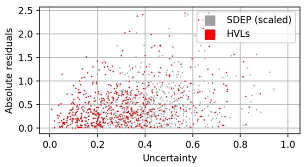

P49146

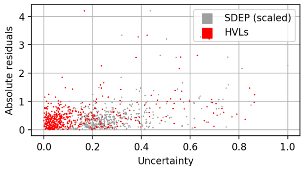

P61169

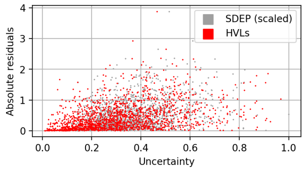

Q05397

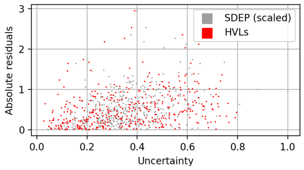

Q16602

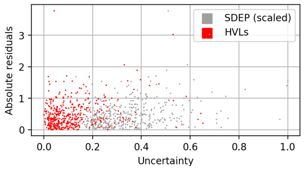

P24941

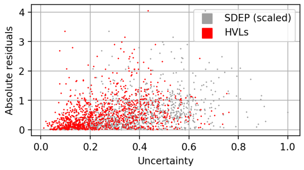

Q92731

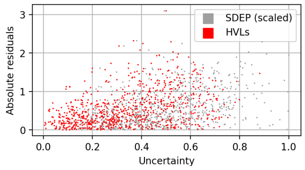

TETRAH

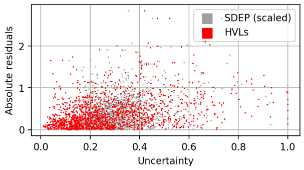

DELANEY

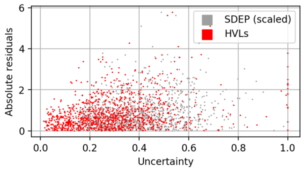

FREESOLV

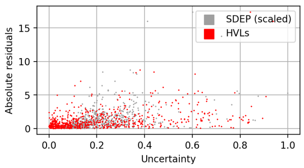

9 SDEP vs. HVLs Scatter Plots (RDKit descriptors)

Table S7.1: SDEP vs. HVLs scatter plots with Pearson correlation coefficient of each data set using RDKit descriptors 1–16.

F7	IL4	MMP2	O60674
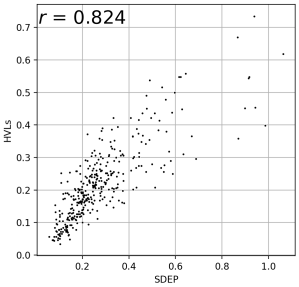	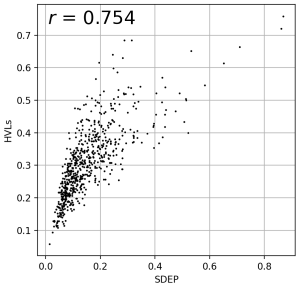	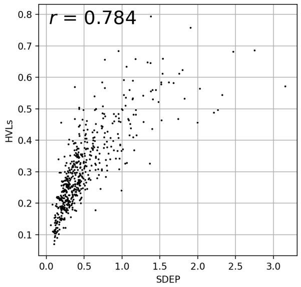	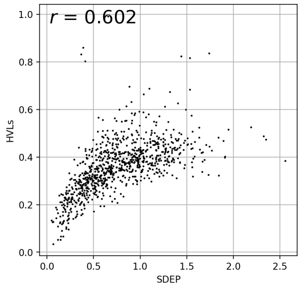
**O14965**	**P03372**	**P04150**	**P06401**
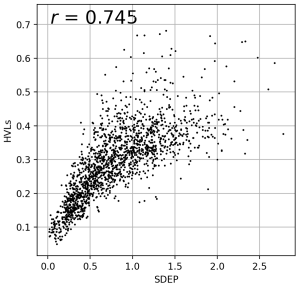	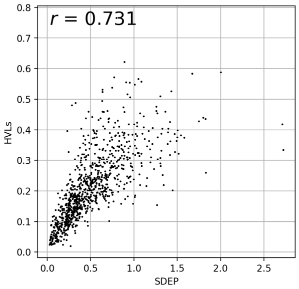	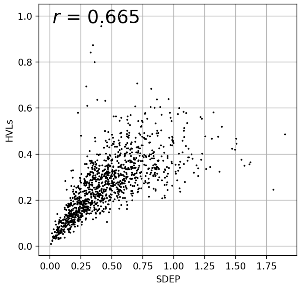	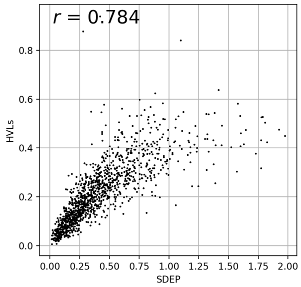
**P11229**	**P12931**	**P16581**	**P17252**
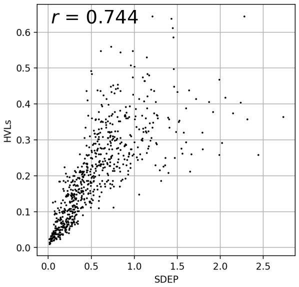	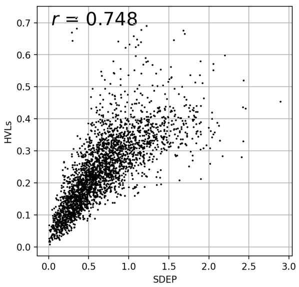	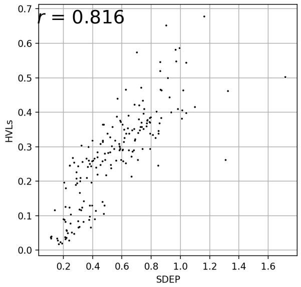	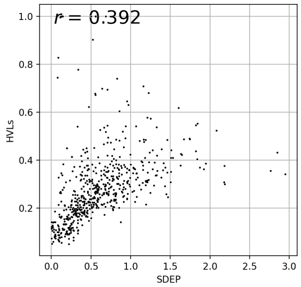
**P18089**	**P19327**	**P21554**	**P24530**
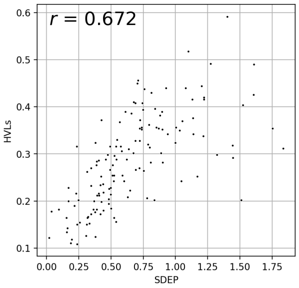	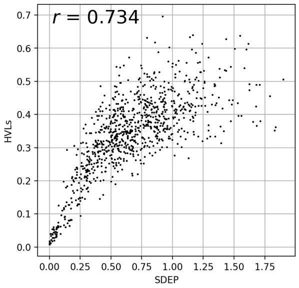	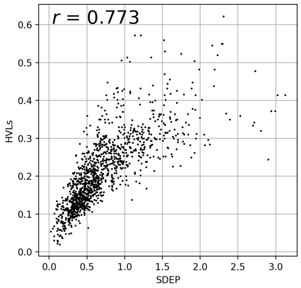	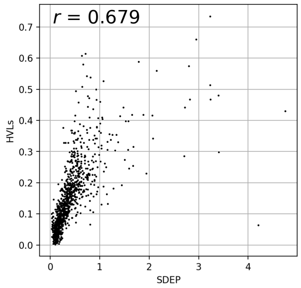

Table S7.2: SDEP vs. HVLs scatter plots with Pearson correlation coefficient of each data set using RDKit descriptors 17–32.

P25929	P28335	P28482	P35968
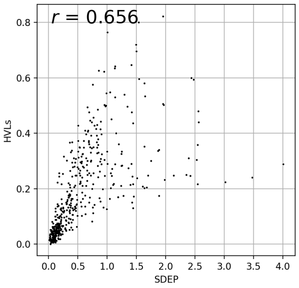	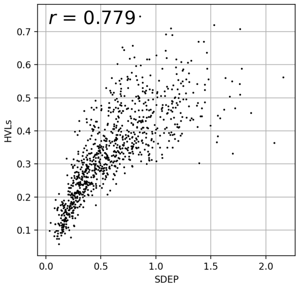	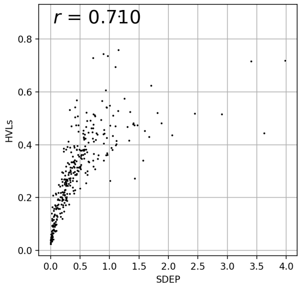	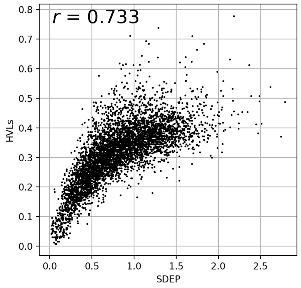
P41594	P42345	P47871	P49146
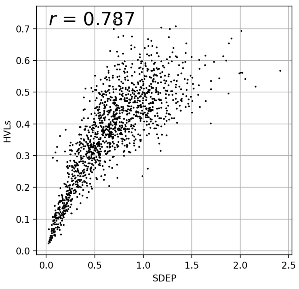	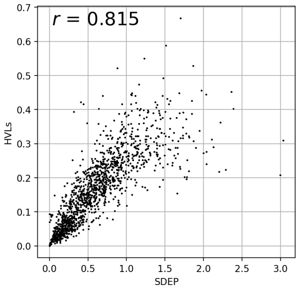	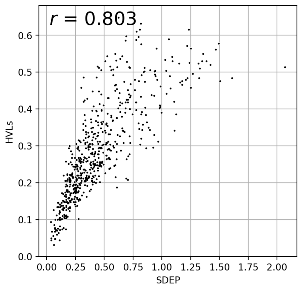	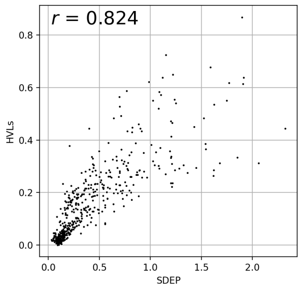
P61169	Q05397	Q16602	P24941
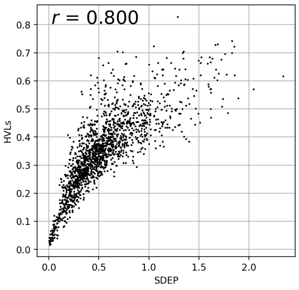	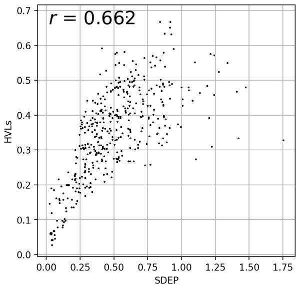	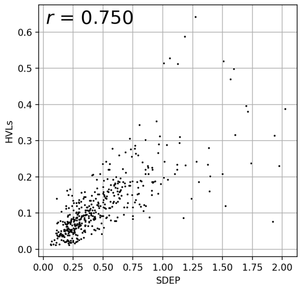	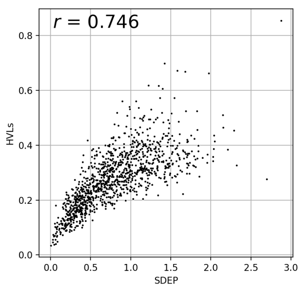
Q92731	TETRAH	DELANEY	FREESOLV
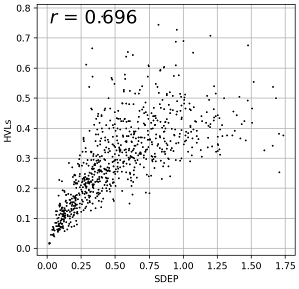	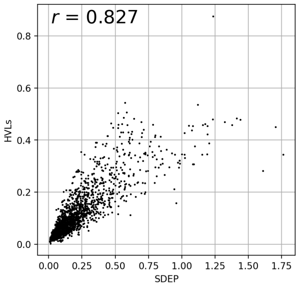	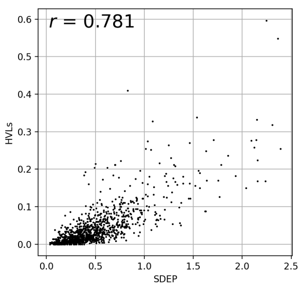	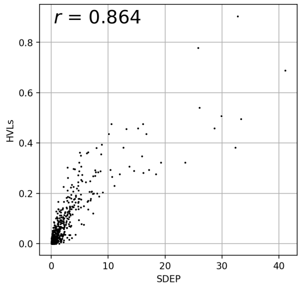

10 SDEP vs. HVLs Scatter Plots (ECFPs)

Table S8.1: SDEP vs. HVLs scatter plots with Pearson correlation coefficient of each data set using ECFPs 1–16.

F7	IL4	MMP2	O60674
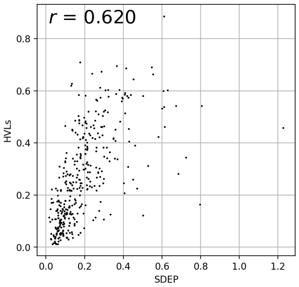	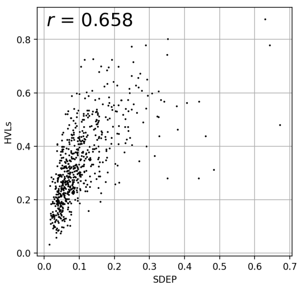	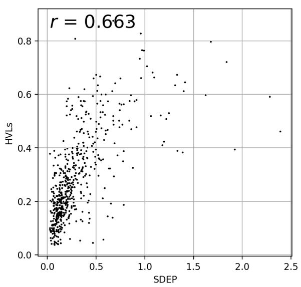	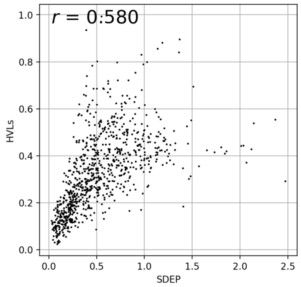
**O14965**	**P03372**	**P04150**	**P06401**
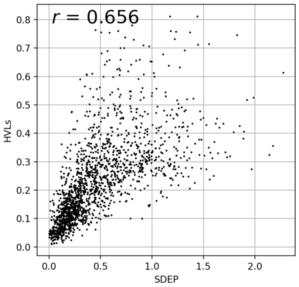	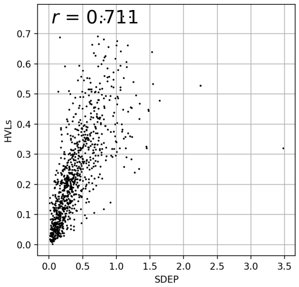	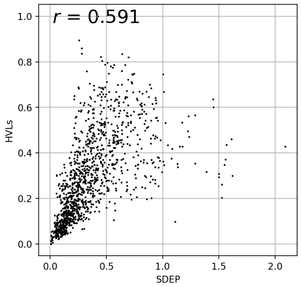	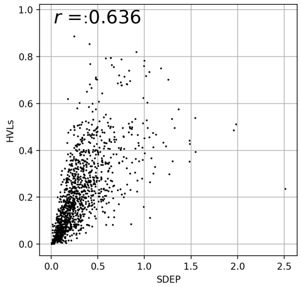
**P11229**	**P12931**	**P16581**	**P17252**
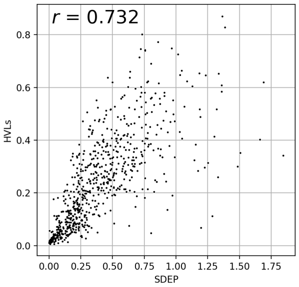	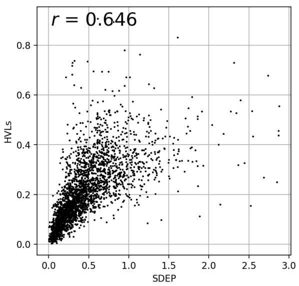	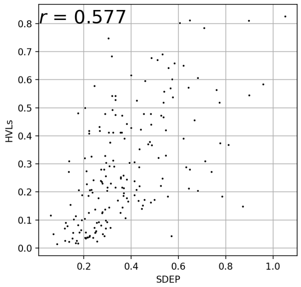	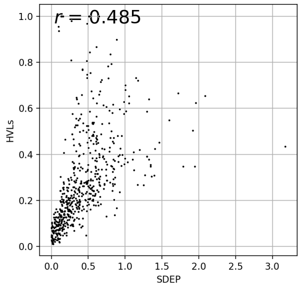
**P18089**	**P19327**	**P21554**	**P24530**
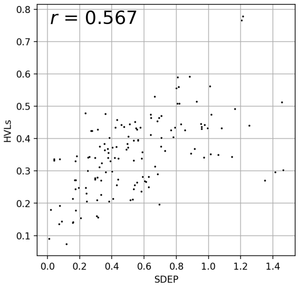	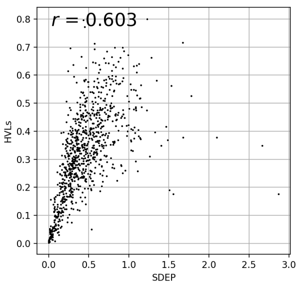	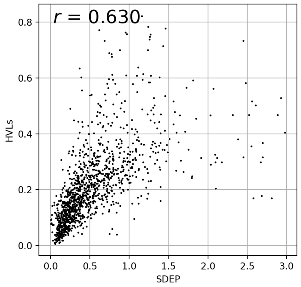	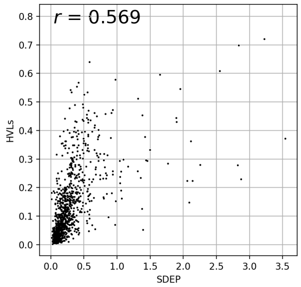

Table S8.2: SDEP vs. HVLs scatter plots with Pearson correlation coefficient of each data set using ECFPs 17–32.

P25929	P28335	P28482	P35968
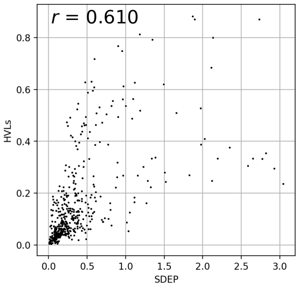	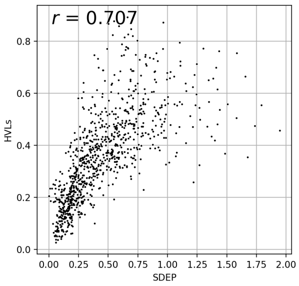	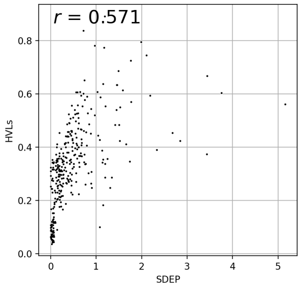	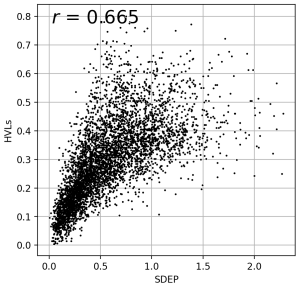
**P41594**	**P42345**	**P47871**	**P49146**
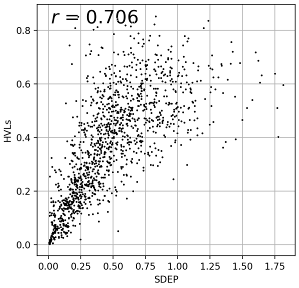	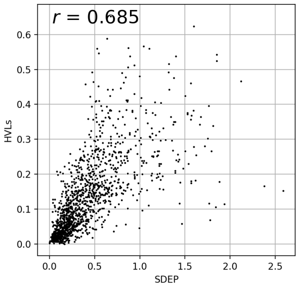	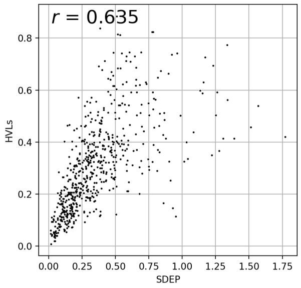	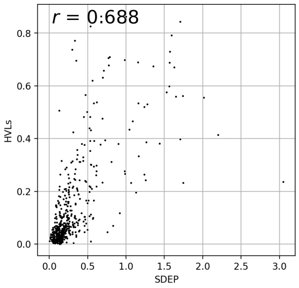
**P61169**	**Q05397**	**Q16602**	**P24941**
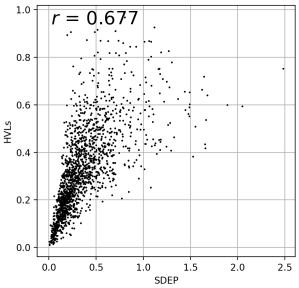	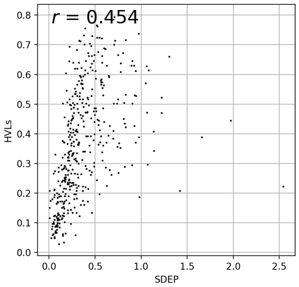	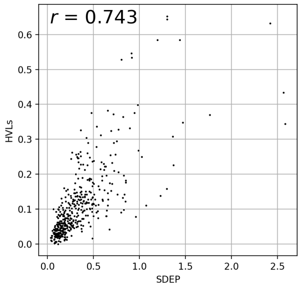	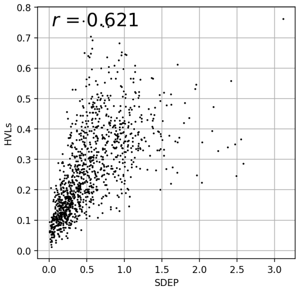
**Q92731**	**TETRAH**	**DELANEY**	**FREESOLV**
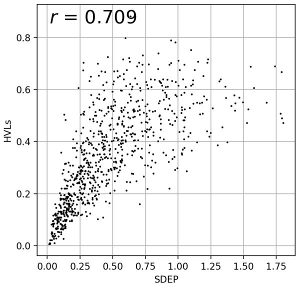	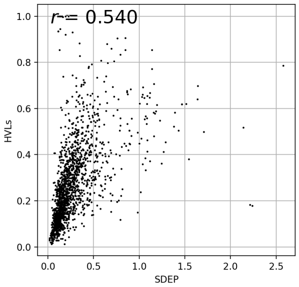	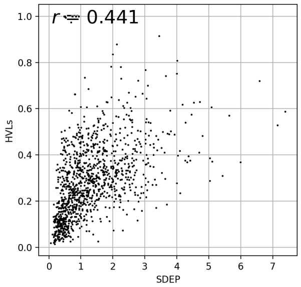	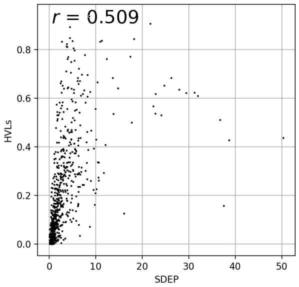

11 $AUCO_{50}$ and MSE Decline (RDKit Descriptors, SDEP)

Table S9: $AUCO_{50}$ and declining MSE for all data sets, using RDKit descriptors and SDEP.

Data set	$AUCO_{50}$	MSE_0	MSE_5	MSE_{10}	MSE_{20}	MSE_{50}	MSE_{90}
F7	23.247	0.277	0.257	0.250	0.240	0.215	0.087
IL4	20.655	0.146	0.129	0.120	0.112	0.095	0.061
MMP2	33.814	0.465	0.376	0.302	0.251	0.186	0.221
O60674	133.538	0.742	0.685	0.665	0.606	0.499	0.539
O14965	153.792	0.610	0.529	0.489	0.422	0.296	0.162
P03372	83.438	0.489	0.443	0.399	0.362	0.219	0.103
P04150	112.030	0.444	0.405	0.380	0.356	0.270	0.152
P06401	85.871	0.399	0.352	0.319	0.278	0.192	0.094
P11229	61.879	0.500	0.467	0.407	0.364	0.249	0.236
P12931	272.810	0.546	0.497	0.460	0.412	0.286	0.177
P16581	25.140	0.620	0.612	0.611	0.552	0.423	0.234
P17252	64.855	0.539	0.468	0.452	0.409	0.228	0.145
P18089	19.145	0.606	0.612	0.565	0.510	0.373	0.233
P19327	110.471	0.565	0.535	0.514	0.475	0.308	0.083
P21554	125.804	0.556	0.508	0.458	0.412	0.323	0.152
P24530	63.339	0.368	0.298	0.284	0.258	0.205	0.135
P25929	26.791	0.473	0.359	0.336	0.260	0.099	0.054
P28335	93.487	0.519	0.493	0.472	0.428	0.291	0.117
P28482	18.929	0.404	0.309	0.292	0.247	0.165	0.008
P35968	540.635	0.648	0.595	0.551	0.499	0.377	0.216
P41594	152.160	0.664	0.610	0.579	0.516	0.364	0.065
P42345	139.026	0.534	0.461	0.445	0.405	0.255	0.096
P47871	46.600	0.451	0.416	0.382	0.324	0.260	0.155
P49146	30.333	0.399	0.326	0.274	0.236	0.187	0.074
P61169	152.533	0.498	0.441	0.406	0.373	0.253	0.067
Q05397	38.583	0.484	0.445	0.429	0.413	0.276	0.192
Q16602	34.572	0.439	0.361	0.352	0.302	0.262	0.273
P24941	131.991	0.661	0.602	0.551	0.494	0.350	0.112
Q92731	77.095	0.496	0.460	0.443	0.393	0.253	0.140
TETRAH	40.312	0.190	0.145	0.122	0.099	0.052	0.010
DELANEY	86.361	0.413	0.349	0.327	0.273	0.194	0.080
FREESOLV	103.439	1.539	1.113	0.949	0.499	0.142	0.026

12 $AUCO_{50}$ and MSE Decline (RDKit Descriptors, HVLs)

Table S10: $AUCO_{50}$ and declining MSE for all data sets, using RDKit descriptors and HVLs.

Data set	$AUCO_{50}$	MSE_0	MSE_5	MSE_{10}	MSE_{20}	MSE_{50}	MSE_{90}
F7	22.641	0.277	0.266	0.255	0.236	0.203	0.138
IL4	24.799	0.146	0.130	0.125	0.121	0.107	0.074
MMP2	38.490	0.465	0.408	0.348	0.273	0.195	0.117
O60674	166.238	0.742	0.748	0.728	0.685	0.562	0.241
O14965	200.348	0.610	0.577	0.559	0.510	0.293	0.135
P03372	99.156	0.489	0.466	0.459	0.379	0.244	0.098
P04150	121.941	0.444	0.421	0.407	0.371	0.287	0.115
P06401	95.120	0.399	0.368	0.341	0.302	0.203	0.136
P11229	78.875	0.500	0.491	0.474	0.392	0.296	0.254
P12931	317.993	0.546	0.518	0.489	0.447	0.318	0.160
P16581	28.796	0.620	0.601	0.602	0.619	0.490	0.328
P17252	68.019	0.539	0.513	0.486	0.435	0.287	0.106
P18089	22.118	0.606	0.613	0.579	0.484	0.462	0.384
P19327	120.599	0.565	0.536	0.520	0.497	0.363	0.107
P21554	146.456	0.556	0.522	0.490	0.446	0.344	0.227
P24530	69.361	0.368	0.325	0.308	0.274	0.204	0.147
P25929	30.593	0.473	0.426	0.376	0.287	0.095	0.074
P28335	92.455	0.519	0.502	0.481	0.426	0.288	0.162
P28482	22.302	0.404	0.357	0.322	0.258	0.150	0.005
P35968	664.302	0.648	0.626	0.602	0.561	0.418	0.216
P41594	156.891	0.664	0.618	0.588	0.547	0.356	0.071
P42345	157.067	0.534	0.510	0.490	0.434	0.287	0.110
P47871	54.629	0.451	0.422	0.399	0.372	0.265	0.154
P49146	31.028	0.399	0.365	0.321	0.239	0.143	0.072
P61169	159.241	0.498	0.458	0.420	0.377	0.253	0.055
Q05397	47.408	0.484	0.479	0.466	0.438	0.338	0.149
Q16602	47.311	0.439	0.434	0.401	0.379	0.324	0.290
P24941	155.641	0.661	0.638	0.611	0.561	0.383	0.141
Q92731	84.324	0.496	0.483	0.460	0.410	0.275	0.129
TETRAH	49.817	0.190	0.161	0.138	0.107	0.063	0.028
DELANEY	95.285	0.413	0.372	0.325	0.296	0.204	0.084
FREESOLV	129.184	1.539	1.144	1.060	0.625	0.337	0.153

13 $AUCO_{50}$ and MSE Decline (ECFPs, SDEP)

Table S11: $AUCO_{50}$ and declining MSE for all data sets, using ECFPs and SDEP.

Data set	$AUCO_{50}$	MSE_0	MSE_5	MSE_{10}	MSE_{20}	MSE_{50}	MSE_{90}
F7	23.430	0.303	0.291	0.254	0.235	0.185	0.236
IL4	19.635	0.133	0.120	0.113	0.104	0.097	0.053
MMP2	43.898	0.389	0.318	0.276	0.252	0.189	0.275
O60674	106.529	0.597	0.549	0.520	0.467	0.368	0.221
O14965	127.627	0.449	0.386	0.363	0.306	0.202	0.137
P03372	86.834	0.497	0.460	0.422	0.353	0.231	0.103
P04150	112.982	0.445	0.418	0.400	0.354	0.285	0.185
P06401	96.436	0.373	0.345	0.303	0.285	0.188	0.103
P11229	80.072	0.522	0.509	0.469	0.393	0.262	0.114
P12931	265.985	0.478	0.429	0.399	0.339	0.258	0.105
P16581	22.759	0.539	0.544	0.511	0.521	0.422	0.463
P17252	58.800	0.495	0.462	0.388	0.366	0.198	0.031
P18089	21.649	0.640	0.629	0.613	0.581	0.408	0.362
P19327	99.933	0.481	0.465	0.448	0.404	0.309	0.084
P21554	122.959	0.513	0.458	0.438	0.390	0.300	0.135
P24530	54.497	0.306	0.247	0.240	0.208	0.170	0.108
P25929	26.764	0.386	0.332	0.255	0.195	0.136	0.044
P28335	89.905	0.499	0.472	0.461	0.385	0.251	0.144
P28482	19.949	0.393	0.316	0.281	0.237	0.099	0.016
P35968	512.414	0.544	0.507	0.478	0.426	0.304	0.168
P41594	140.017	0.561	0.527	0.493	0.448	0.303	0.136
P42345	111.891	0.450	0.386	0.372	0.337	0.211	0.122
P47871	51.888	0.421	0.396	0.365	0.318	0.263	0.154
P49146	27.130	0.423	0.347	0.281	0.220	0.154	0.162
P61169	141.103	0.417	0.381	0.356	0.311	0.225	0.129
Q05397	38.735	0.471	0.442	0.404	0.389	0.250	0.101
Q16602	33.883	0.416	0.377	0.366	0.296	0.236	0.320
P24941	139.801	0.581	0.522	0.502	0.438	0.312	0.097
Q92731	78.244	0.516	0.472	0.454	0.408	0.274	0.170
TETRAH	92.841	0.291	0.261	0.245	0.215	0.175	0.120
DELANEY	354.877	1.278	1.243	1.186	1.093	0.788	0.346
FREESOLV	460.930	4.516	3.559	2.904	2.277	1.176	0.150

14 $AUCO_{50}$ and MSE Decline (ECFPs, HVLs)

Table S12: $AUCO_{50}$ and declining MSE for all data sets, using ECFPs and HVLs.

Data set	$AUCO_{50}$	MSE_0	MSE_5	MSE_{10}	MSE_{20}	MSE_{50}	MSE_{90}
F7	26.504	0.303	0.306	0.291	0.290	0.160	0.115
IL4	21.868	0.133	0.120	0.116	0.108	0.101	0.054
MMP2	43.355	0.389	0.343	0.303	0.274	0.181	0.139
O60674	117.224	0.597	0.576	0.559	0.518	0.350	0.198
O14965	148.103	0.449	0.413	0.381	0.342	0.199	0.125
P03372	98.829	0.497	0.479	0.430	0.392	0.227	0.118
P04150	115.344	0.445	0.430	0.403	0.362	0.261	0.169
P06401	94.472	0.373	0.344	0.317	0.282	0.186	0.117
P11229	86.207	0.522	0.514	0.492	0.452	0.267	0.152
P12931	294.809	0.478	0.452	0.432	0.382	0.271	0.159
P16581	18.223	0.539	0.534	0.506	0.487	0.358	0.456
P17252	60.816	0.495	0.463	0.443	0.404	0.201	0.094
P18089	25.350	0.640	0.647	0.611	0.647	0.483	0.409
P19327	112.308	0.481	0.457	0.455	0.445	0.313	0.060
P21554	127.030	0.513	0.487	0.455	0.402	0.253	0.158
P24530	61.991	0.306	0.288	0.264	0.232	0.171	0.138
P25929	33.780	0.386	0.353	0.319	0.213	0.185	0.033
P28335	91.531	0.499	0.471	0.443	0.406	0.264	0.205
P28482	23.343	0.393	0.339	0.283	0.248	0.158	0.041
P35968	574.535	0.544	0.530	0.505	0.454	0.310	0.177
P41594	142.865	0.561	0.536	0.500	0.449	0.274	0.112
P42345	126.405	0.450	0.416	0.385	0.349	0.230	0.152
P47871	54.811	0.421	0.406	0.376	0.335	0.260	0.121
P49146	31.747	0.423	0.363	0.291	0.243	0.161	0.212
P61169	143.340	0.417	0.401	0.380	0.329	0.204	0.099
Q05397	49.958	0.471	0.472	0.464	0.411	0.320	0.161
Q16602	43.134	0.416	0.362	0.352	0.330	0.340	0.374
P24941	161.951	0.581	0.582	0.573	0.515	0.314	0.259
Q92731	82.619	0.516	0.509	0.482	0.430	0.238	0.145
TETRAH	93.066	0.291	0.272	0.252	0.211	0.157	0.127
DELANEY	328.800	1.278	1.205	1.081	1.000	0.823	0.342
FREESOLV	454.261	4.516	3.345	2.979	2.347	1.495	0.719

15 Summary of all Data Sets

Table S13: All data sets used for evaluation. The original number of compounds refers to the number of measurement points in the raw files.

Label	Category	Original no. compounds	No. compounds after filtering	Dependent variable	Output unit
F7	Activity	365	357	Inhibitory concentration	pIC_{50}
IL4	Activity	665	647	Inhibitory concentration	pIC_{50}
MMP2	Activity	549	540	Inhibitory concentration	pIC_{50}
O60674	Activity	869	869	Inhibitory concentration	pIC_{50}
O14965	Activity	1651	1651	Inhibitory concentration	pIC_{50}
P03372	Activity	908	908	Inhibitory concentration	pIC_{50}
P04150	Activity	1182	1182	Inhibitory concentration	pIC_{50}
P06401	Activity	1233	1233	Inhibitory concentration	pIC_{50}
P11229	Activity	843	683	Inhibitory concentration	pIC_{50}
P12931	Activity	2719	2719	Inhibitory concentration	pIC_{50}
P16581	Activity	184	184	Inhibitory concentration	pIC_{50}
P17252	Activity	580	580	Inhibitory concentration	pIC_{50}
P18089	Activity	137	137	Inhibitory concentration	pIC_{50}
P19327	Activity	1018	880	Inhibitory concentration	pIC_{50}
P21554	Activity	1392	1216	Inhibitory concentration	pIC_{50}
P24530	Activity	1030	955	Inhibitory concentration	pIC_{50}
P25929	Activity	501	467	Inhibitory concentration	pIC_{50}
P28335	Activity	926	896	Inhibitory concentration	pIC_{50}
P28482	Activity	322	322	Inhibitory concentration	pIC_{50}
P35968	Activity	4662	4662	Inhibitory concentration	pIC_{50}
P41594	Activity	1381	1285	Inhibitory concentration	pIC_{50}
P42345	Activity	1337	1337	Inhibitory concentration	pIC_{50}
P47871	Activity	944	599	Inhibitory concentration	pIC_{50}
P49146	Activity	561	486	Inhibitory concentration	pIC_{50}
P61169	Activity	1968	1622	Inhibitory concentration	pIC_{50}
Q05397	Activity	416	416	Inhibitory concentration	pIC_{50}
Q16602	Activity	660	431	Inhibitory concentration	pIC_{50}
P24941	Activity	1130	1130	Inhibitory concentration	pIC_{50}
Q92731	Activity	799	799	Inhibitory concentration	pIC_{50}
TETRAH	Non-activity	1571	1571*	Inhibitory growth concentration	$pIGC_{50}$
DELANEY	Non-activity	1144	1144*	Aqueous solubility	log mol/L
FREESOLV	Non-activity	643	643*	Hydration free energy	kcal/mol

*Activity data set filtering steps were not applied.

16 Dependent Variable Histograms

Table S14.1: Distribution of the dependent variables of each data set as histograms 1–16.

F7

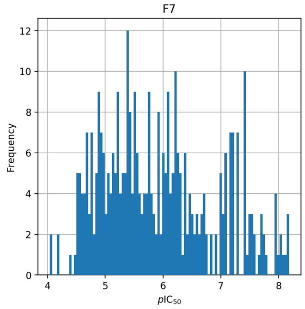

IL4

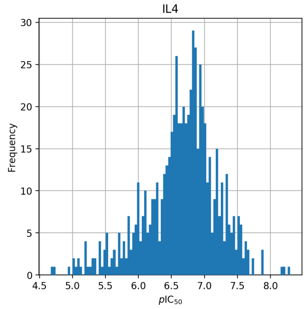

MMP2

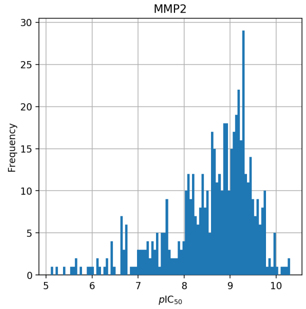

O60674

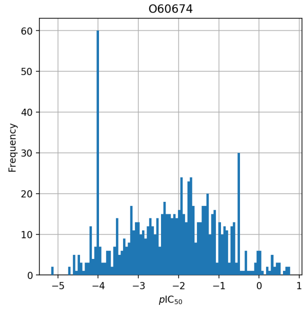

O14965

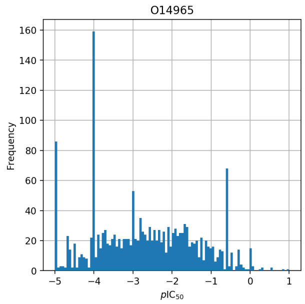

P03372

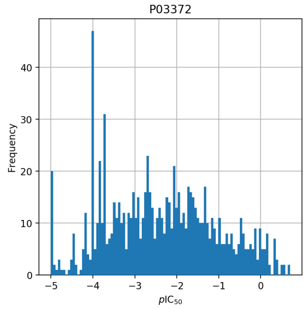

P04150

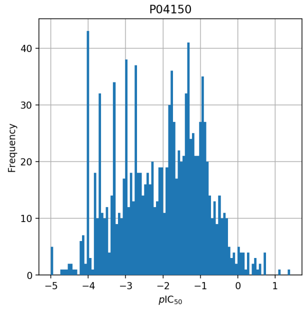

P06401

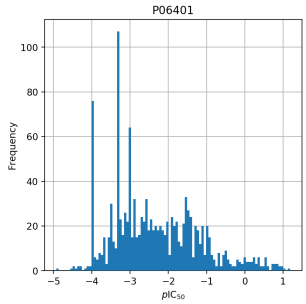

P11229

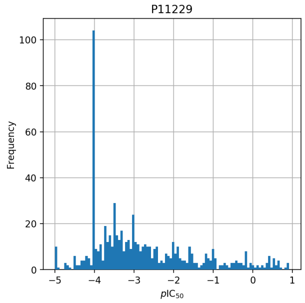

P12931

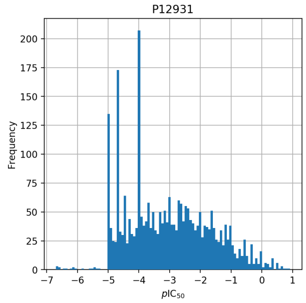

P16581

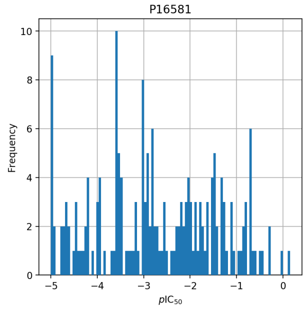

P17252

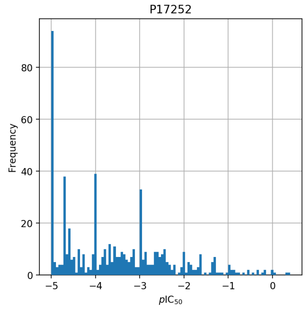

P18089

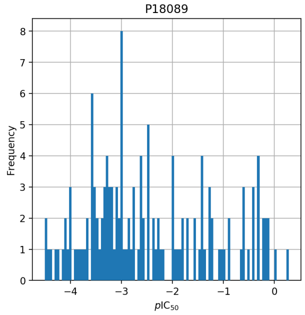

P19327

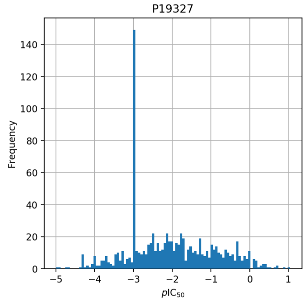

P21554

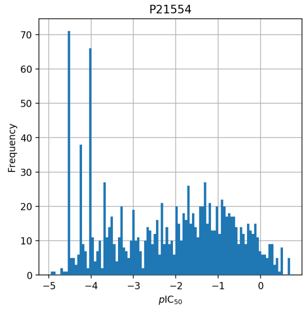

P24530

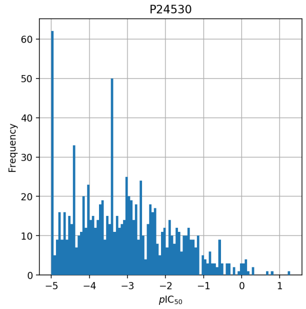

Table S14.2: Distribution of the dependent variables of each data set as histograms 17–32.

P25929	P28335	P28482	P35968
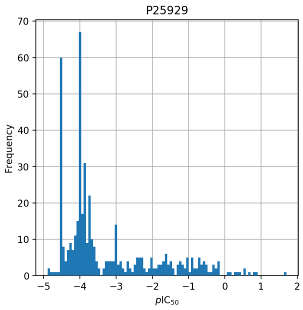	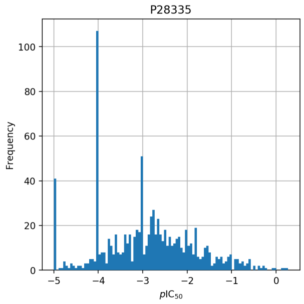	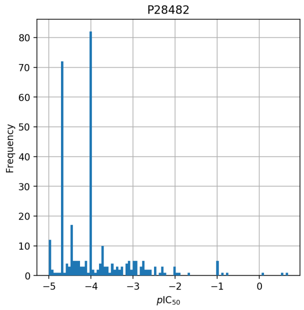	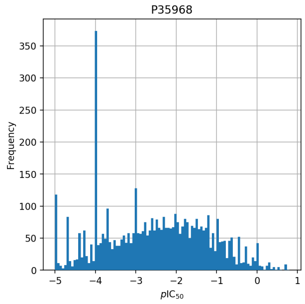
**P41594**	**P42345**	**P47871**	**P49146**
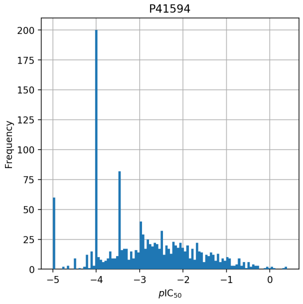	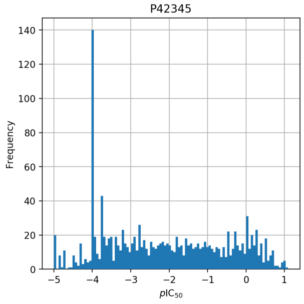	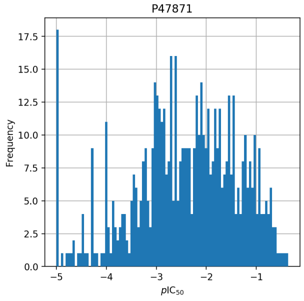	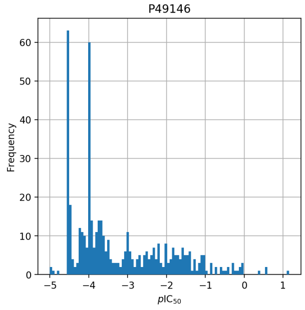
**P61169**	**Q05397**	**Q16602**	**P24941**
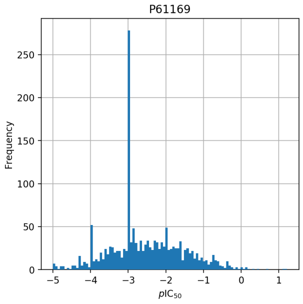	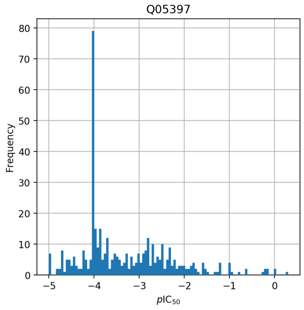	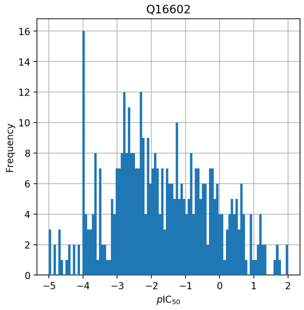	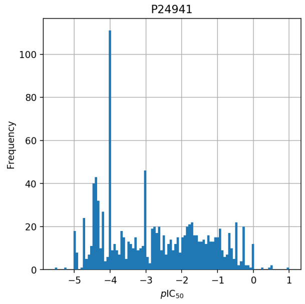
**Q92731**	**TETRAH**	**DELANEY**	**FREESOLV**
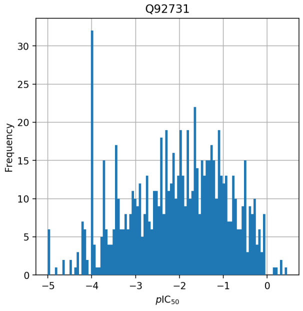	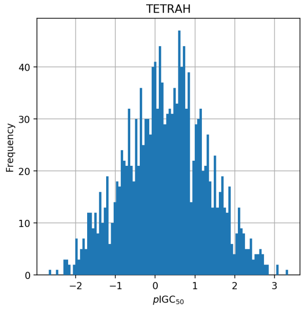	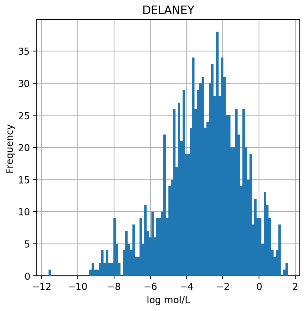	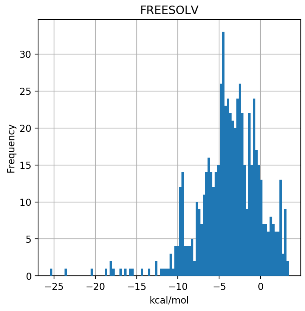

17 Python Packages with Versions

Table S15: Python packages with versions required to run the framework.

Package	Version
Python itself	3.7.3
Matplotlib	3.4.3
NumPy	1.21.2
pandas	1.3.4
scikit-learn	0.24.2
tqdm	4.61.0
